# Comprehensive assessment, review, and comparison of AI models for solar irradiance prediction based on different time/estimation intervals

**DOI:** 10.1038/s41598-022-13652-w

**Published:** 2022-06-10

**Authors:** Olusola Bamisile, Dongsheng Cai, Ariyo Oluwasanmi, Chukwuebuka Ejiyi, Chiagoziem C. Ukwuoma, Oluwasegun Ojo, Mustapha Mukhtar, Qi Huang

**Affiliations:** 1 Sichuan Industrial Internet Intelligent Monitoring and Application Engineering Technology Research Centre, Chengdu University of Technology, Chenghua District, Chengdu, Sichuan People’s Republic of China; 2 School of Software Engineering, University of Electronic Science and Technology of China, Chengdu, Sichuan People’s Republic of China; 3 IMDEA Networks Institute, 28918 Leganes, Madrid, Spain; 4 Universidad Carlos III de Madrid, 28912 Leganes, Madrid, Spain; 5 School of Economics and Management, Guangdong University of Petrochemical Technology, Maoming, 525000 China

**Keywords:** Environmental sciences, Energy science and technology, Engineering

## Abstract

Solar energy-based technologies have developed rapidly in recent years, however, the inability to appropriately estimate solar energy resources is still a major drawback for these technologies. In this study, eight different artificial intelligence (AI) models namely; convolutional neural network (CNN), artificial neural network (ANN), long short-term memory recurrent model (LSTM), eXtreme gradient boost algorithm (XG Boost), multiple linear regression (MLR), polynomial regression (PLR), decision tree regression (DTR), and random forest regression (RFR) are designed and compared for solar irradiance prediction. Additionally, two hybrid deep neural network models (ANN-CNN and CNN-LSTM-ANN) are developed in this study for the same task. This study is novel as each of the AI models developed was used to estimate solar irradiance considering different timesteps (hourly, every minute, and daily average). Also, different solar irradiance datasets (from six countries in Africa) measured with various instruments were used to train/test the AI models. With the aim to check if there is a universal AI model for solar irradiance estimation in developing countries, the results of this study show that various AI models are suitable for different solar irradiance estimation tasks. However, XG boost has a consistently high performance for all the case studies and is the best model for 10 of the 13 case studies considered in this paper. The result of this study also shows that the prediction of hourly solar irradiance is more accurate for the models when compared to daily average and minutes timestep. The specific performance of each model for all the case studies is explicated in the paper.

## Introduction

Nowadays, the world is almost impossible to envisage without its interrelationship and dependence on electricity^1^. This electricity is mainly produced with fossil fuels and based on statistics, the global primary energy demand will increase by over 59% between 2002 and 2030 ^2^. However, the evidential environmental impact of the current (fossil fuels) energy resources, as well as the need to reduce its climate change effect, led to the development of renewable energy sources (RES) ^3^. These RES have experienced significant growth in recent decades and they are projected to have as much as 39% share in global electricity generation by 2050 ^4^. Solar energy is a sustainable, clean, and extremely abundant RES ^5^ that poses a very low risk to its immediate environment and the world at large. The critical investigation into the accessibility and availability of renewable energy (RE) resources has witnessed a continuous evolvement, especially in developing countries. There is a rapid and consistent escalation in electricity demand in many developing countries as they strive toward advanced technological implementation and globalization ^6^. Therefore, it is imperative to initiate and encourage RES development in these regions.

Solar radiation influences agricultural production, atmospheric circulation, hydrological processes, public health as well as ecological services, and the comprehensive knowledge of this parameter at any location is important to its environmental sustainability and economic potential ^7^. Moreover, solar radiation is a crucial and decisive parameter for solar energy management and generation. Information about global solar radiation is also significant in many applications including; RE-usage, hydrology, and meteorology ^8^. The recent efforts and push for the replacement of fossil fuels with RES have made solar radiation a more important meteorological variable used to simulate and measure RE potential in any location. Unlike other meteorological parameters like relative humidity, temperature, and sunshine duration, the observation stations for solar radiation measurement are not globally available. This is due to the complicated measurement techniques and relatively high cost. Therefore, developing an accurate method or model to predict solar radiation is very important ^9^.

Typically, the models for solar radiation prediction or estimation can be classified into empirical, statistical, physical, and machine learning models ^9^. While physical models such as sky-image-based models explore the mechanism between solar radiation and other meteorological parameters ^10^, empirical models are aimed at developing a linear or non-linear regression equation for solar radiation estimation ^11^. Statistical models such as the autoregressive moving-average model (ARIMA), are developed based on statistical correlation ^12^. In recent years, artificial intelligence (AI) models have been used for better solar radiation prediction. The ability of these models to simulate nonlinear and complex relationship mapping as well as the capability to learn and extract meaning features from the input data via backpropagation and parameter update make it more desirable for this task ^13^.

The adoption of AI (machine learning and deep learning) models for the prediction or estimation of solar radiation have proven in literature to have a wider application and higher accuracy in comparison to other models. These models can accurately moderate the long-term, medium-term, and short-term prediction of solar radiation ^14^. Gurel et al. ^15^ presented the assessment of time series (Holt-Winters), machine learning (feed-forward neural network), empirical models (3 Angstrom-type models), and response surface methodology (RSM) for global solar radiation. Meteorological data obtained between 2008 and 2018 for four provinces in Turkey were used to train, validate, and test the models. Based on the performance evaluation of their models, the R^2^ varied between 0.952 and 0.993 while the artificial neural network was concluded to present the best results ^15^. Furthermore, a review of some of the most recent literatures on solar radiation prediction with different models and methods is summarized in Table 1. This table highlights the type of model, case study, the aim of the study, and the performance summary of the models in different works of literature. Based on the articles reviewed in this table, the use of both unsupervised (machine) learning and supervised learning algorithms has been proposed for the forecast of solar irradiance. Therefore, the comparison of these models is one of the aims of this present study. Also, none of the proposed models were able to give a 100% accurate prediction/forecast of solar radiation in all the various locations. Hence the consistent recommendation stated in most of these research articles that future studies are required in this research domain to develop more accurate models for solar radiation forecasting.

**Table 1:** Summary of recent literature on solar radiation forecast/prediction.

Author/References	Case study	Research objective	Models used	Performance of models
Sun et al.^16^	Beijing China	Improvement of the performance of solar radiation forecasting and comparison with other models	Decomposition-clustering-ensemble learning	NRSME = 2.96% MAPE = 2.83% Directional forcast = 88.24%
Belmahdi et al.^17^	Tetouan city Morocco	Building models that can forecast monthly mean daily global radiation	Time series (ARMA and ARIMA)	ARIMA (0.2,1) gave a better performance than ARMA (2,1) with 64.05% and 24.32% improvement respectively
Blal et al.^18^	Adrar Algeria	Statistically comparing the predictive models used for daily average global radiation estimation and hourly global solar radiation study on the horizontal surface under different weather conditions (Studying solar radiation under various conditions of climate)	Six Ambient temperature models	Model (M4) gave R^2^ of 0.8753 being best M1 = 0.7099 M5 = 0.8193
Heng et al. ^19^	United States	The model used for forecasting with accuracy and stability objective for global monthly average radiation	nondominated sorting-based multiobjective bat algorithm (NSMOBA)	Gave satisfactory accuracy and stability
Kisi et al. ^20^	Turkey	Connectionist system evolution for daily scale prediction of solar radiation	Dynamic evolving neural-fuzzy inference system (DENFIS)	Provided better accuracy in monthly SR prediction than the benchmark models
Ghimire et al. ^21^	Australia	Integration of CNN and LSTM for short-term GSR prediction	hybrid model based on a convolution network CLSTM	Performed better than other DL models and the benchmark models
Rodríguez-Benítez et al. ^22^	Spain	Extension of a temporal horizon of ASI-based nowcast to match the satellite-based prediction. Increasing the temporal latency and resolution of the satellite-based nowcasting to match that of ASI-based prediction	all-sky imager (ASI) model	ASIs are preferable to other models since it overcomes most challenges that other models encounter
Peng et al. ^23^	Alabama USA	Construction and evaluation of the performance of DL models based on biLSTM, SCA, and CEEMDAN for hourly solar radiation prediction over multi-step horizons	deep learning model based on Bi-directional long short-term memory (BiLSTM), sine cosine algorithm (SCA), and complete ensemble empirical mode decomposition with adaptive noise (CEEMDAN) which can be called CEN-SCA-BiLSTM model	CEN-SCA-BiLSTMmodel gave the smallest RMSE, MAE, MASE, and largest R when compared with other competitors
Campo-Ávila et al. ^24^	Spain	Prediction of one day ahead hourly global solar radiation	A model that combines clustering, regression, and classification	RMSE less than 20%
Lai et al. ^25^	Brazil	Hourly solar forecasting with Feature Attention-based Deep Forecasting (FADF)	A deep learning-based hybrid method	RMSE 11.88% on Itupiranga dataset and 12.65% on Ocala dataset when compared with smart persistence
Guermoui et al. ^26^	Algeria	multi-step ahead forecasting of daily global and direct horizontal solar radiation components in the Saharan climate	Weighted Gaussian Process Regression (WGPR),	RMSE = 3.18 and R^2^ = 85.85% for 10^th^ daily global horizontal radiation and RMSE = 5.23 and R^2^
Gürel et al. ^15^	Turkey	Using four different models to predict monthly average daily global SR data	ML algorithm-based models	R^2^ = 0.952 ~ 0.993 RMSE and MAPE less than 10%
Zhuo et al. ^27^	China	To simultaneously predict the multi-time scale (daily and monthly mean daily) and multi-component (global and diffuse) solar radiation	combined multi-task learning and Gaussian process regression (MTGPR) model	Average R^2^ ranges 0.19 ~ 0.48%, RMSE improved 0.57 ~ 0.65% and rRMSE improved 0.51% ~ 0.52% for daily prediction. For monthly prediction the range is 2.62 ~ 2.65%, 5.50 ~ 12.07% and 5.21 ~ 12.08% respectively for R^2^, RMSE and rRMSE
Makade et al. ^28^	India	Developing a comprehensive review of the works done by Indian researchers in solar radiation modeling and carrying out a statistical analysis of the developed solar radiation model	GSR Model M-78	MPE varies between -8.1186% and 6.9383% and the coefficient of determination between 0.6345 and 0.9616
Prasad et al. ^29^	Australia	Development of a hybrid model that handles issues with nonstationarity in multiple predictor inputs utilizing a self-adaptive approach while giving a good accuracy of the forecast of short-term	multivariate empirical mode decomposition method (MEMD) – Singular Value Decomposition (SVD)- Random Forest (RF) model (hybrid MEMD-SVD-RF model)	Generated a better and more reliable forecast Average R^2^ of 0.98 and RMSE of 1.05
Z. Pung et al. ^30^	Alabama US	To study the performances of DL algorithms for the prediction of solar radiation	An ANN model and a recurrent neural network (RNN) model	RNN model improved by 47% in NMBE and 26% in RMSE
Puah et al. ^31^	Malaysia	Producing a comparable forecast performance in relation with the Supervised Learning	Regression Enhanced Incremental Self-organising Neural Network (RE-SOINN)	Achieved higher accuracy when compared to others MASE = 0.65755 RMSE = 73.945
Narvaez et al. ^32^	Colombia	Develo[ping accurate site-adaptation as well as solar radiation model using ML and DL	ML-based model	38% better performance than the traditional methods
Karaman et al. ^33^	Karaman Turkey	Using different activation functions to obtain the best response from ELM and ANN after their performance has been compared	extreme learning machines (ELM) and Artificial Neural Network (ANN)	ELM has better performance with RMSE = 0.0297 and Performance of 95%
A˘gbulut et al. ^34^	Turkey	Prediction of daily global solar radiation from 4 different provinces having diverse solar radiation distribution	support vector machine (SVM), artificial neural network (ANN), kernel and nearest-neighbor (k-NN), and deep learning (DL) models	R^2^ ranges from 85.5%—93.6% MAPE 15.92%—30.24% rRMSE 14.10%—25.19%
Al-Rousan et al. ^35^	Jordan	Reviewing different prediction methods employed in predicting solar radiation	Multi-layer perceptron (MLP), Support Vector Machine Regression (SVMR), and Linear regression (LR)	R^2 =^ 0.9513, 0.8477 and 0.8477 respectively for MLP, SVMR and LR while MAPE = 0.0001, 0.0418 and 0.0434
Sunhra Das ^36^	India	To carry out short term solar forecasting for different days of the year	A model for prediction of solar radiation on tilted surface	RMSE = 8.9, 6.7, and 8.3 for Jan 29th, Apr 1st, and Oct 6th respectively
Bounoua et al. ^37^	Morocco	Evaluation of the potential of three ensemble methods based on regression trees (Bagging, Boosting, and RandomForest) in estimating the daily GHI	empirical and machine-learning methods	Random Forest method with the following result R: 87.53–96.20%; nMAE: 5.84–11.81%; nRMSE: 7.85–15.33% outperformed others
Shadab et al. ^38^	India	extending the ARIMA models for spatial forecasting of monthly average insolation as well as finding the most suitable location for solar power projects based on the forecasts	Seasonal ARIMA (SARIMA) model	R^2^ = 0.9293, Root Mean Square Error = 0.3529, Mean Absolute Error = 0.2659 and Mean Absolute Percentage Error = 6.556
Srivastava et al. ^39^	India	forecasting of the 1-day-ahead to 6-day-ahead solar radiation levels using four ML models	MARS, CART, M5 and random forest models	Random Forest provided the best result while the Cart has the worst result. From best to worst we have Random Forest > M5 > MARS > CART

The expansion of solar energy-based technologies and applications will continue ^40^. Therefore, the reliable estimation of solar radiation including its hourly, daily average, monthly average, annual, ^41^ and seasonal variability is of paramount importance for the estimation of solar energy capacity and potential ^42^. As mentioned earlier, the high cost and technological complexity attached to the measurement of solar radiation makes it a more difficult task in many meteorological stations. For example, there are 1798 meteorological stations in Turkey in the year 2020 and only 129 of the stations are capable of measuring solar radiation ^43^. Also, out of the 756 meteorological stations in China, only 122 of them have the capability to measure solar radiation ^44^. These further stresses the importance of solar radiation estimation. In most existing works of literature on solar radiation prediction, the prediction was done with different models. However, these models were compared based on the similarity of the class. Also, most models are used to predict a particular type of data type with a specific timestep. This has raised research questions about the adoption of different models for the various dataset, timesteps, and locations. Furthermore, developing countries (especially Africa) have enormous solar energy potential, however, the development of solar-based technologies has been very slow due to many reasons. One of which is inadequacies in the measurements of solar radiation.

Therefore, in this paper, we seek to further the knowledge of literature in this field by comparing different artificial intelligence (AI) models for solar radiation estimations. Eight different AI models namely; convolutional neural network (CNN), artificial neural network (ANN), long short-term memory recurrent model (LSTM), eXtreme gradient boost algorithm (XG Boost), multiple linear regression (MLR), polynomial regression (PLR), decision tree regression (DTR), and random forest regression (RFR) are compared for solar irradiance forecast. Additionally, two hybrid deep neural network models are developed in this study for this task. These models are a combination of two or more deep neural network models namely; ANN-CNN and CNN-LSTM-ANN. In comparison to existing techniques where a specific timestep is adopted, in this study, the models developed will be used to estimate the hourly, every minute, and daily average solar radiation. Also, different datasets such as typical meteorological year (TMY), surface radiation data set for heliostats (SARAH), and The World Bank solar radiation measurement data (WB-ESMAP) dataset are used to test the models developed in this paper. In comparison to literature where a specific solar irradiance data set is used, the research further contributes to literature by considering different measured solar irradiance datasets. These datasets include; global beam direct solar irradiance (GSR), diffused solar irradiance (DSR), daily average solar radiation flux at the surface normal to the direction of the sun (DNI), global horizontal irradiance measured from silicon pyranometer (GHI_Sil_), diffused horizontal irradiance from rotating shadowband irradiometer (DHI_RSI_), and global horizontal irradiance measured from thermopile pyranometer (GHI_pyr_). These are useful for solar photovoltaics, solar thermal, solar heliostat, solar rooftop, and other solar technology applications.

This study seeks to determine the AI model that has a consistent accurate predictive performance for solar irradiance measured with various methods in different locations. Therefore, the datasets used in this study have been collected from 13 specific locations across six African countries. The viability of different AI models, when used for solar radiation prediction in different locations and considering various datasets as well as timesteps, is analysed in this study. One of the research questions that this study seeks to address is the possible sovereignty of an AI model for solar radiation estimation tasks considering differences in location, timestep, and dataset. While developing (African) countries has been used as the case study for the implementation of the AI algorithms developed in this study, the applicability of this models is not limited to developing countries only. They can be use in developed countries also however, some of the training parameters may require adjustments for the supervised AI algorithm. The rest of the article is organized as follows; a brief introduction to all the models considered in this study as well as the model development are explained in “Machine learning and deep learning algorithms” and “Data acquisition and preparation” sections. The performances of the models are presented in “Results” section and a brief discussion of these performances is stated in “Brief summary and discussion” section. The entire article is concluded in “Conclusions” section.

## Machine learning and deep learning algorithms

Recent research have focused on forecasting renewable energy resources ^45–47^, because of the growth in global RES and the integration of such sources into the electrical grid throughout the world. Recently, the projection of renewable energy production, notably wind and solar energy, has received considerable attention due to its considerable influence on operating and managing power management choices. Precise forecasts for the production of renewable energy-based systems are essential to ensure the continued dependability of the grid and to decrease energy market and energy systems risks/costs. Due to nature, the energy generated by solar and wind energies will always be unstable. Hence, the need to adopt sophisticated methodologies for the forecast of energy systems’ production. The methods adopted and compared in this study for solar energy resources forecast may be divided into 4 categories: physical methods, statistical models, techniques, and hybrid ways of artificial intelligence ^48^. These are introduced in the following subsection.

### Random forest regression

**Figure 1:**
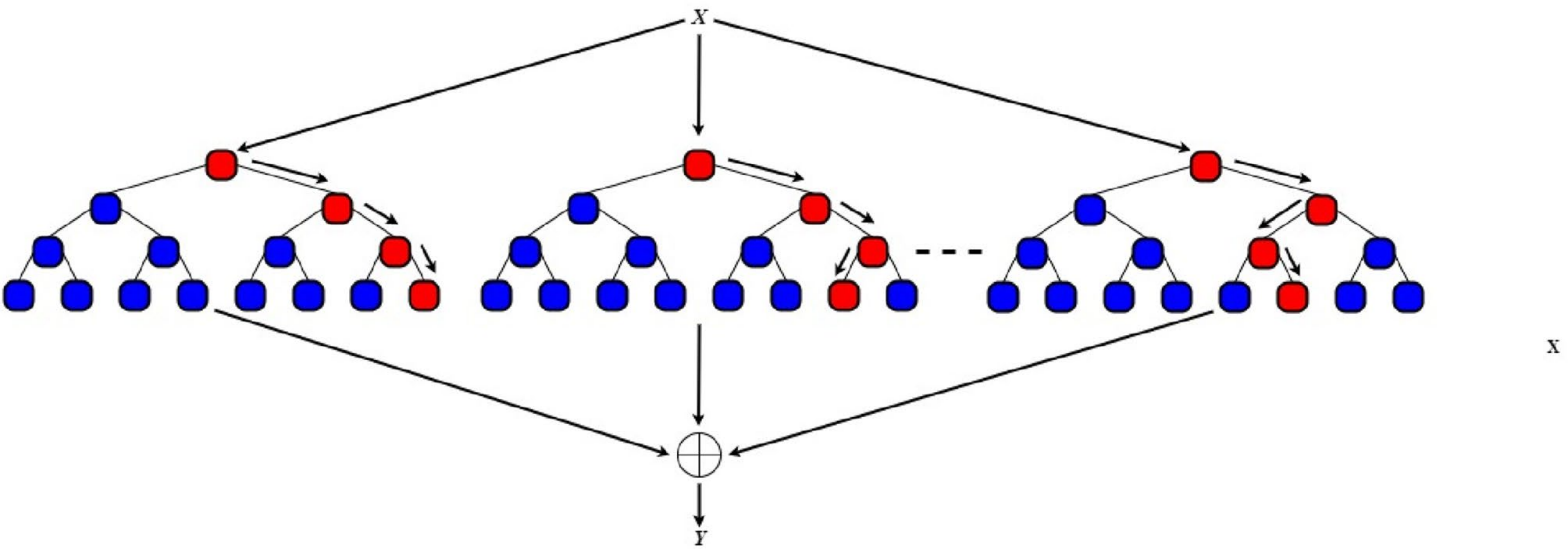
Sample of a random forest tree.

One of the most common machine learning methods is a random forest (RF) algorithm ^49^. This is a controlled approach that employs a regression method for learning. The learning approach integrates various machine learning algorithms in order to generate predictions that are more accurate than a single model. In the course of training and determining the mean class of the classes, a random forest operates by building many decision trees as a forecast for all the trees ^50,51^. Creating several trees for different subsets of the data points balances the prevalent overfitting problem, minimizes variance, and ensures improved accuracy. The RF algorithm is shown in Algorithm 1 while a sample of the RF tree is illustrated in Fig. 1.

**Table:** 

**Algorithm 1** Random Forest Algorithm
Start
Select from the training set a random k data point
Construct a decision tree for the k data points
Select N of the trees you would want to construct
Repeat
Steps 1 and 2
Make a prediction of the values of y for the data point for each of your N-trees and assign the new data point to the average across the whole number of y-values anticipated
End

The RF algorithm's predictive value is provided by the mathematical equation ^52^;

1 $$ \hat{Y} = \frac{1}{N}\mathop \sum \limits_{n = 1}^{N} T_{n} \left( X \right) $$

where $Y$'s mean values are from $n, N, and T_{n} \left( x \right).$ Input parameters in $X$ indicate the number of random forest decision trees in N. The equation specifies the average number of $T_{n} , n = 1,2,...,N$ decision trees given the input $X$ in order to provide a solid forecast.

With the RF-Method, forecasts can be obtained and forecasting parameters identified (which are related to the response) via RF's integrated measurement of variable importance. This may also be taken into consideration and enhanced prognostics can be produced. Specifically, RF is adopted in this study for solar radiation forecast due to its use in existing works of literatures ^53^. For instance, in three distinct sites with varied API conditions in China, Sun et al. ^54^ utilize the random forest to estimate solar radiation given a single, accessible meteorological variable and air pollution index.

### Polynomial regression

Polynomial regression is a specialized linear regression in which the data (having a curvilinear connection between the goal and the independent variables) are multinomially equated. Polynomial ensures a proper approximation of dependent and independent variables across a wide range of curvatures. The value of the target variable does not vary uniformly with regard to the predictor in a curvilinear relationship (s). The linear regression equation (Eq. (2)) with one predictor is transformed to polynomial equation of degree n in polynomial regression as Eq. (3).

2 $$ Y = \theta_{0} + \theta_{1} x $$

where $Y$ is the goal, $x$ is the predictor, $\theta_{0}$ is the bias, and $\theta_{1}$ is the weight of the equation of regression.

3 $$ Y = \theta_{0} + \theta_{1} x + \theta_{2} x^{2} + \theta_{3} x^{3} + \ldots + \theta_{n} x^{n} $$

Here $\theta_{0}$ is the bias, $\theta_{0} , \theta_{1} , \ldots . \theta_{n}$ are the weight of the polynomial regression equation and $n$ is the polynomial degree. Since hourly solar radiation profile follows a polynomial path, this AI algorithm is modelled in this study for the forecast of solar irradiance in accordance with the literature ^55^.

### Multi-linear regression

This AI algorithm employs numerous explanatory factors to predict the result of the response variable. The objective of multiple linear regression (MLR) model is to describe the linear connection between the (independent) explanatory and the (dependent) responsive variables. The connection of many independent variables $\left( {x_{1} ,x_{2} ,x_{3} ,x_{4} } \right)$ and a dependent variable $\left( {\hat{y}} \right)$ is explored and the first order of regression function employed in this investigation is presumed to be;

4 $$ \hat{y} = b_{0} + b_{1} x_{1} + b_{2} x_{2} + b_{3} x_{3} + b_{4} x_{4} $$

where $$b_{0}$$ is the y-axis cut-off point for the adjusted regression curve, $$b_{1}$$ is the first variable of guess $$x_{1}$$, and $$b_{2}$$ is the first variable of guessing $$x_{2}$$. The independent variables; wind speed, temperature, humidity, and pressure ($$x_{1}$$, $$x_{2}$$, $$x_{3} $$ and $$x_{4}$$) and dependency variable ($$\hat{y}$$) solar radiation are correspondingly used as a in this study.

### Decision tree regression

**Figure 2:**
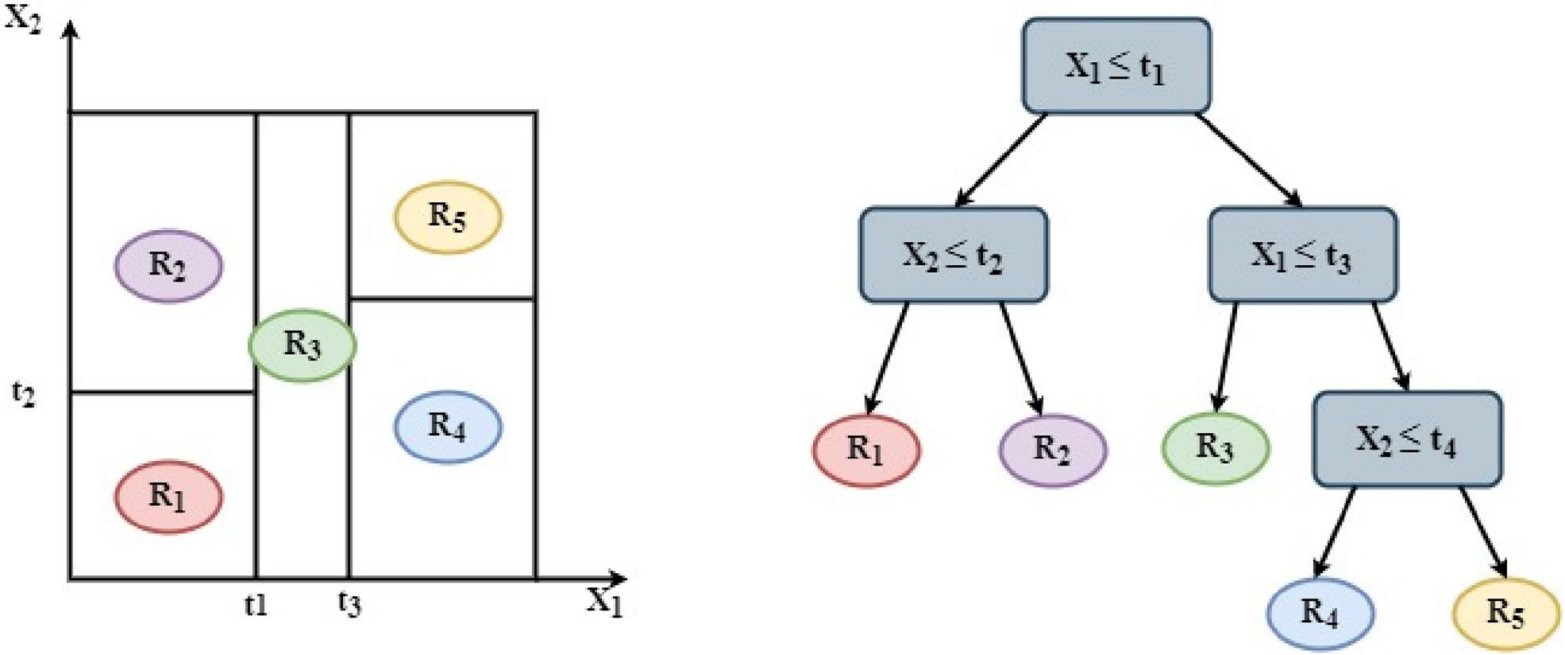
Schematic representation of regression tree.

Decision trees are hierarchical non-parametric structures, which build both regression and classification models in a tree shape. A decision tree operates recursively and splits the original input space constantly into sub-sets to accumulate instances in smaller areas ^56^. The decision-making tree is gradually created during the breaking process, and a final decision-making tree with leaf nodes is generated. A blade node shows a choice on a discreet or ongoing objective. The ID3 and C4.5 decision tree algorithms, invented by Ross Quinlan, are frequently utilized in literature ^57^. A novel application of decision tree classifier in solar irradiance prediction was presented by Singh et al. ^58^. In this work, the technique of the C4.5 decision tree regression is used because of the continuous nature of the sun irradiance values ^59^. In the form of a model regression tree, a predictor space is divided into j regions $\left( {R_{1} , R_{2} ,R_{3} \ldots ..R_{J} } \right)$ is depicted as Fig. 2. For all instances in the same region, the same prediction is made by the means of answers (for all training examples in the region). The basic goal throughout the construction of a decision tree regression model is to locate regions $\left( {R_{1} , \ldots ..R_{J} } \right)$ which minimize the remaining square sum.

### XG BOOST

eXtreme Gradient Boosting (XG-Boost or XGB) is one of the most recent machine learning algorithms that is very good for 1D dataset. In terms of precision and speed, it has the best performance for most tasks ^60^. It runs in parallel and distributed computing, thereby achieving a higher learning rate in comparison with other set algorithms. XG-boost is a modified algorithm for generalized gradient boosting and it creates a distinct type of tree from the boost algorithm for gradients. The split may be found using a similarity score and gain in XG-boost. The regulating parameter is used to prevent the split from overfitting. When the parameter regularization is nil it falls into the standard technique for gradient boosting. Two more approaches avoid overfitting together with regularization. One is the retraction scales that change the weight by a factor η at each step. Its goal is to decrease an individual tree's effect on the model. The second method is to employ subsampling of columns, which similarly improves training time. Another essential step is that an approximation method is used to identify the optimum division ^61^.

### Long short-term memory (LSTM)

**Figure 3:**
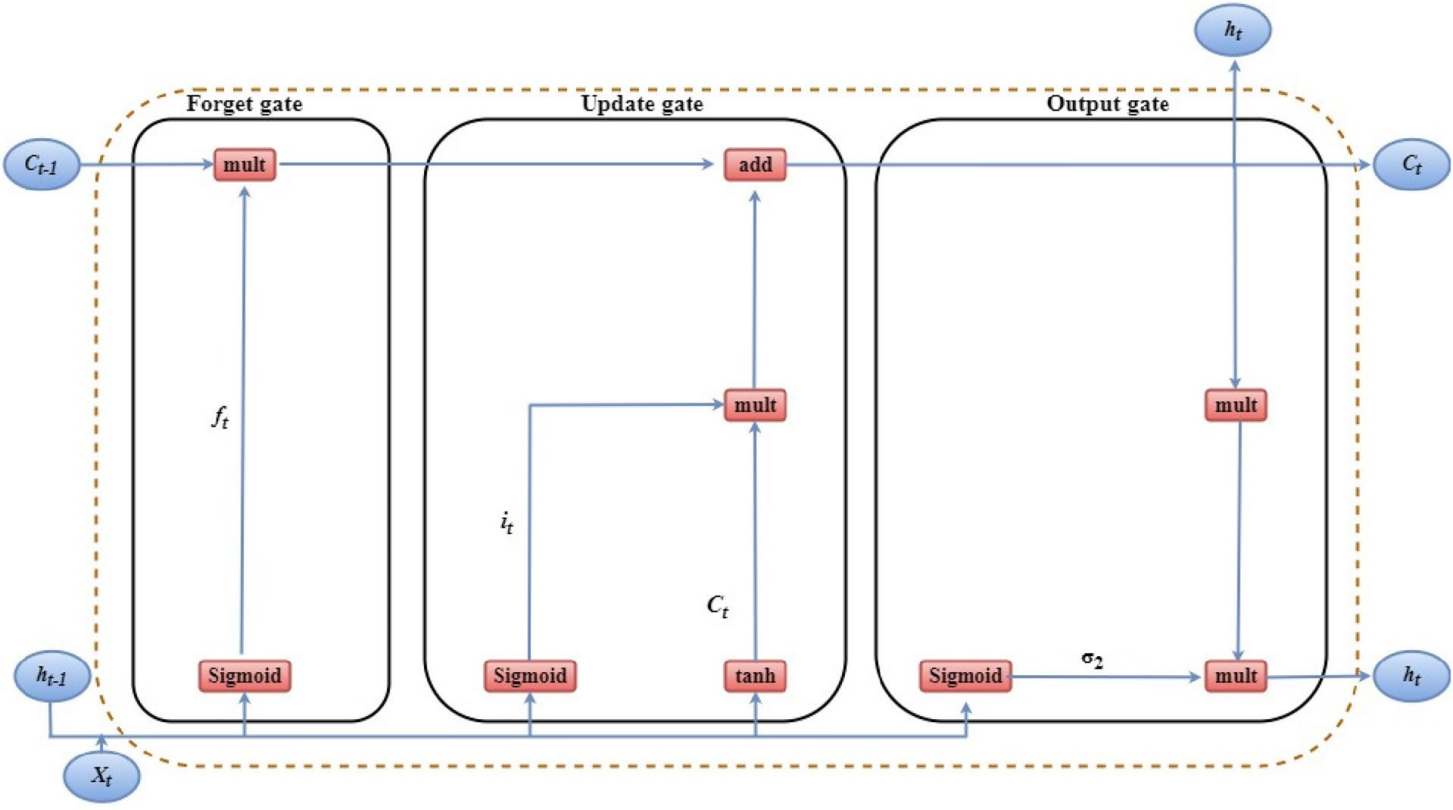
The internal structure of long short-term memory.

For the resolution of the disappearing and exploding gradient problem, LSTM offers memory blocks instead of traditional recurrent neural network (RNN) units ^62^. It then adds a cell state to stored long-term states (Fig. 3) which is the main difference between LSTM and the vanilla RNN. An LSTM network can recall and link prior data to current data ^63^. Three gates are integrated, including the input gate, "forgetful" gate, and output gate where $x_{t}$ references the current input; new and predecessor cell states are referred by $C_{t}$ and $C_{t - 1}$ , respectively; and $h_{t}$ and $h_{t - 1}$ respectively the current and preceding cell outputs. The LSTM input gate principle is expressed in the following forms:

5 $$ i_{t} = \sigma \left( {W_{i} *\left[ {h_{i - 1} , x_{t} } \right] + b_{i} } \right) $$

6 $$ \overset{\lower0.5em\hbox{$\smash{\scriptscriptstyle\smile}$}}{C}_{t} = tanh\left( {W_{i} *\left[ {h_{i - 1} , x_{t} } \right] + b_{i} } \right) $$

7 $$ C_{t} = f_{t} C_{t - 1} + i_{t} \overset{\lower0.5em\hbox{$\smash{\scriptscriptstyle\smile}$}}{C}_{t} $$

where Eq. (5) is utilized to employ a Sigmoid layer to pass $$h_{i - 1}$$ and $$x_{t}$$ to determine the required information. Then $$h_{i - 1}$$ and $$x_{t}$$ passing through the tanh layer in Eq. (6) is used to obtain fresh information. In Eq. (7) $$W_{i}$$ refers to a sigmoid output and $$\overset{\lower0.5em\hbox{$\smash{\scriptscriptstyle\smile}$}}{C}_{t}$$ = a tanh output, the present moment information ($$\overset{\lower0.5em\hbox{$\smash{\scriptscriptstyle\smile}$}}{C}_{t - 1}$$) and the LSTM Information ($$\overset{\lower0.5em\hbox{$\smash{\scriptscriptstyle\smile}$}}{C}_{t}$$) is merged into $$\overset{\lower0.5em\hbox{$\smash{\scriptscriptstyle\smile}$}}{C}_{t}$$ . Here, $$W_{i}$$ indicates weight matrices and $$b_{i}$$ is the LSTM gate bias.

The forgetful gate of the LSTM then permits selective information transmission through a sigmoid layer and a dot product. The choice of forgetting the associated information of an earlier cell with some likelihood, with $W_{f}$ referring to the weight matrix, b_f_ the offset and σ is the sigmoid function, is done using Eq. (8).

8 $$ f_{i} = \sigma \left( {W_{f} *\left[ {h_{t - 1} ,x_{t} } \right] + b_{f} } \right) $$

The output gate of the LSTM determines the state of the following inputs: $h_{t - 1}$ and $x_{t}$ in Eq. (9) and Eq. (13) respectively. The final result is acquired and multiplied through the vectors for state decisions which transmit through the tanh layer new information, C_t_,

9 $$ O_{t} = \sigma \left( {W_{0} *\left[ {h_{t - 1} ,x_{t} } \right] + b_{0} } \right) $$

10 $$ h_{t} = O_{t} \tanh \left( {C_{t} } \right) $$

where $W_{0}$ and $b_{0}$ are the weighted matrices of the output gate and LSTM bias respectively.

### Artificial neural network (ANN)

**Figure 4:**
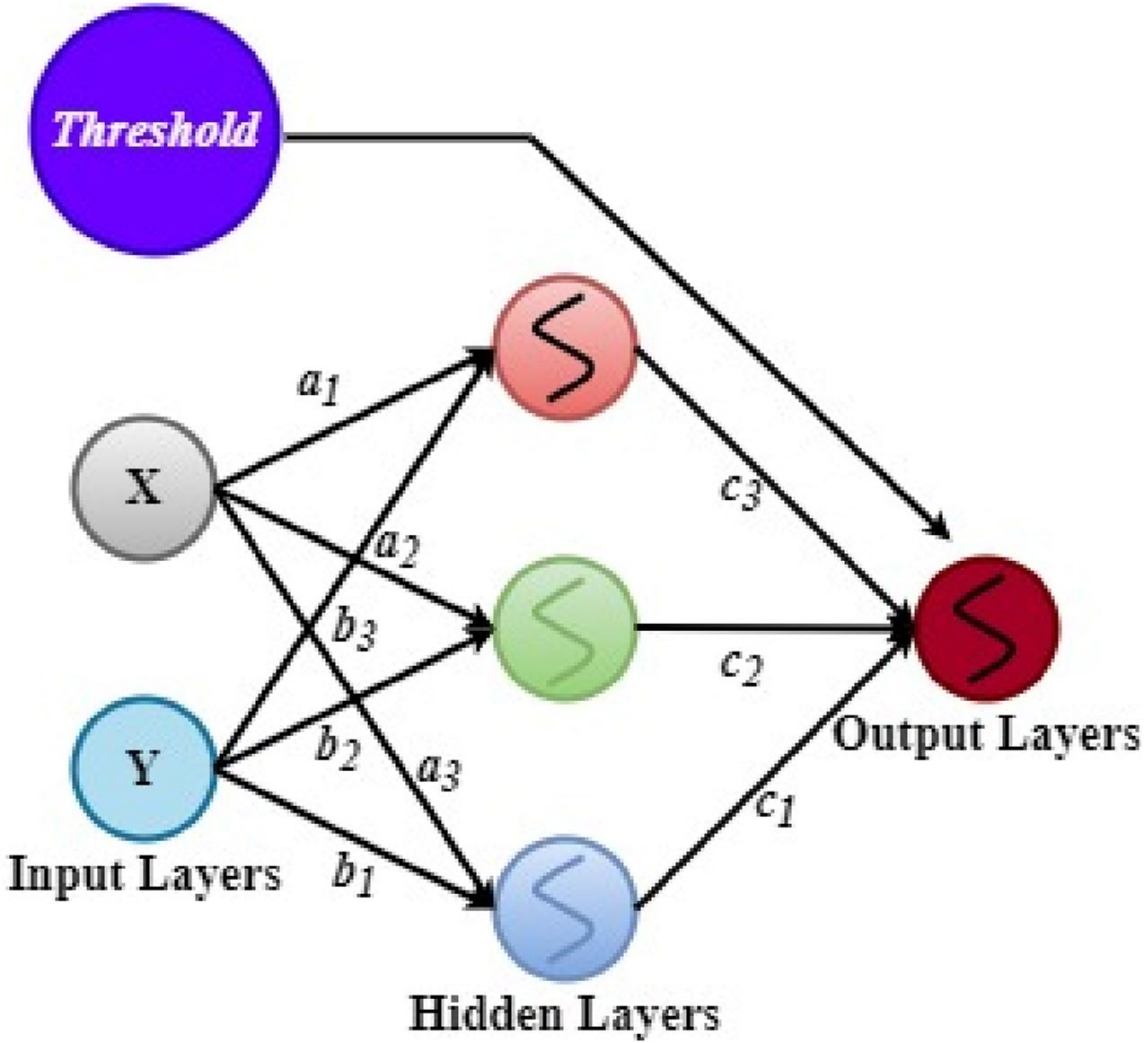
Artificial neural network architecture.

The ANN is an information processing model that imitates biological neural network activities and structures found in human brains ^64^. This AI model is used to solve linear and nonlinear regression tasks. Figure 4 illustrates a basic neural network, with 2 input neurons, X and Y, 3 neurons, and 1 neuron. For the desired offset, the threshold component is utilized. The weights $w_{i,j}$ where the indexes of the neurons are $i and j$ are $a_{i} and b_{i}$. To compute the weighted amount, first X and Y are multiplied by their weights. The result is then added to a partial function and supplied into an activation. Every neuron computed in the hidden layer, $h_{j}$, is calculated with $h_{j} = s\left( {\mathop \sum \limits_{i} w_{i.j} *h_{i} } \right)$, where $S$ is the activation function. The ReLU Rectified Linear Unit (ReLU) function, $S\left( x \right) = {\text{max}}\left( {0, x} \right)$ is used for hidden layer activation and nonlinear activation while the Sigmoid function $S\left( x \right) = \frac{1}{1} + e^{ - x}$ is applied on the output layer to model the network’s probability distribution. ANN is one of the most predominant supervised learning AI algorithm for solar radiation forecast in literature ^65^^–^^67^, hence, its adaptation to the dataset in this study.

### Convolutional neural network (CNN)

**Figure 5:**
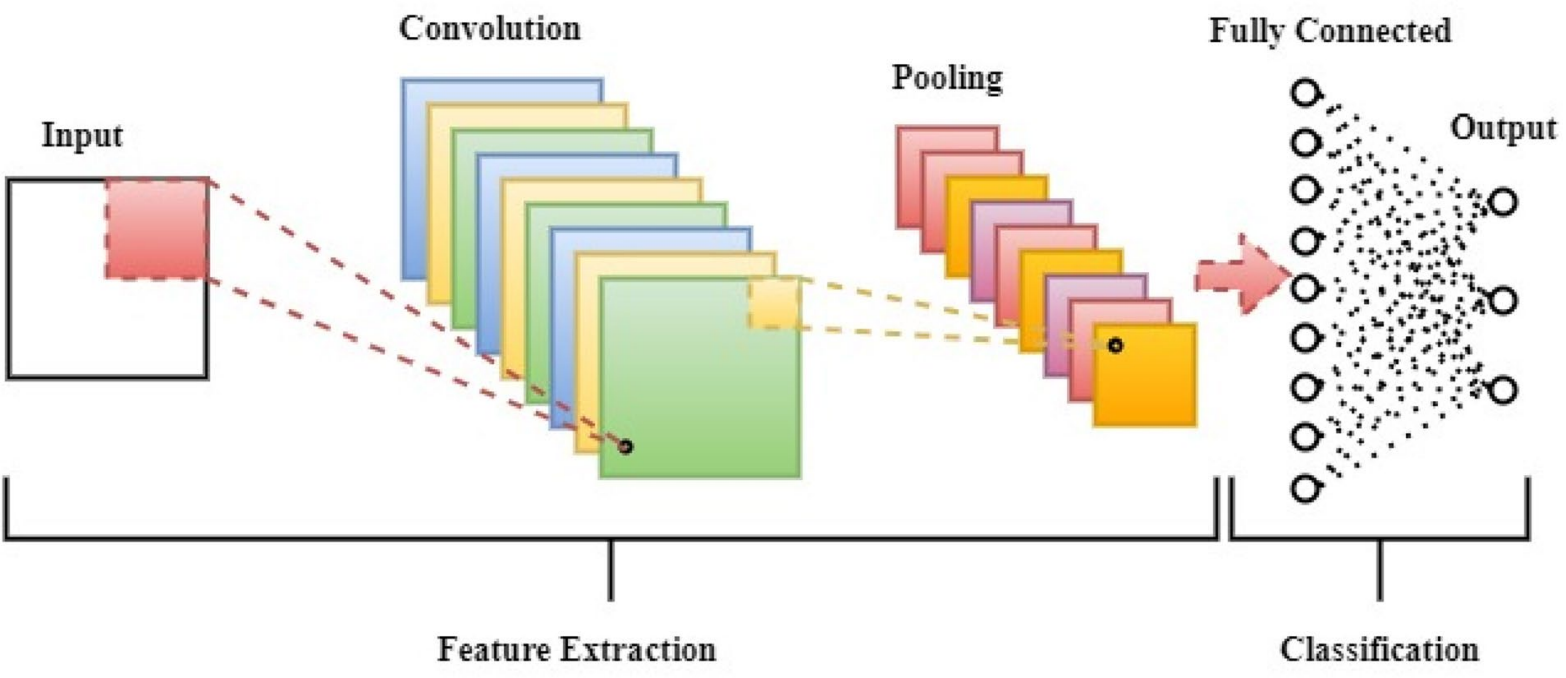
Convolutional neural network architecture.

This model is a special kind of multilayer perceptron, however, unlike other deep learning architecture, the basic neural network is unable to learn complicated characteristics. In several applications ^68^, CNN algorithms have shown great performance in the categorization of images, object recognition, and analysis of medical images. However, it has also been used for solar irradiance prediction tasks in the existing works of literature ^69,70^. The basic principle behind a CNN is that local features are obtained from high layer entrances and transferred for more complicated features to lower layers (as shown in Fig. 5). CNN converts the input data from the input layer into a collection of class scores for the output layer across all linked layers. A CNN includes the full connecting layers, the pooling, and the convolutional layers.

A collection of kernels ^71^ is used to determine the feature mappings tensor in the convolutional layer. These kernels converge a whole input with 'stride(s)' to make a volume in its dimensions ^72^. After the convolutional layer is employed for the processing, the dimensions of an input volume shrink. Therefore, zero-padding ^73^ is necessary for padding input volumes with zeros and maintaining low-level dimensions of an input volume. The functioning of the convolutional layer is:

11 $$ F\left( {i, j} \right) = \left( {I*K} \right)\left( {i, y} \right) = \mathop \sum \nolimits \mathop \sum \nolimits I\left( {i + m,j + n} \right)K\left( {m,n} \right) $$

$$I $$ refers to an input matrix, $$K$$ is a 2D filter of size $$m to n,$$ and $$F$$ is a 2D feature map output. $$I*K$$ indicates the functioning of the convolutionary layer. The rectified linear unit (ReLU) layer is used to increase nonlinearity on feature maps ^74^. By maintaining the threshold input at zero, ReLU calculates the activation. The following is expressed mathematically:

12 $$ f\left( x \right) = {\text{max}}\left( {0,x} \right) $$

Downsampling of a particular dimension is performed by the pooling layer ^75^, in order to minimize parameters. The most frequent way of max-pooling in the input region generates the maximum value. The FC layer ^76^ is utilized as a classifier that decides on the characteristics derived from the convolutions and pooling layers. A CNN aims to learn more about data by use of convolutions. For CNN predictive models it is necessary to collect data from convolutional layers while regression work is carried out in the last fully connected layer ^77^. In this study, the Convolution-1D (Conv1D) which is most suitable for text input data is implemented to convolve the input data points over temporal or single spatial dimensional tensors.

### Hybrid CNN-ANN architecture

**Figure 6:**
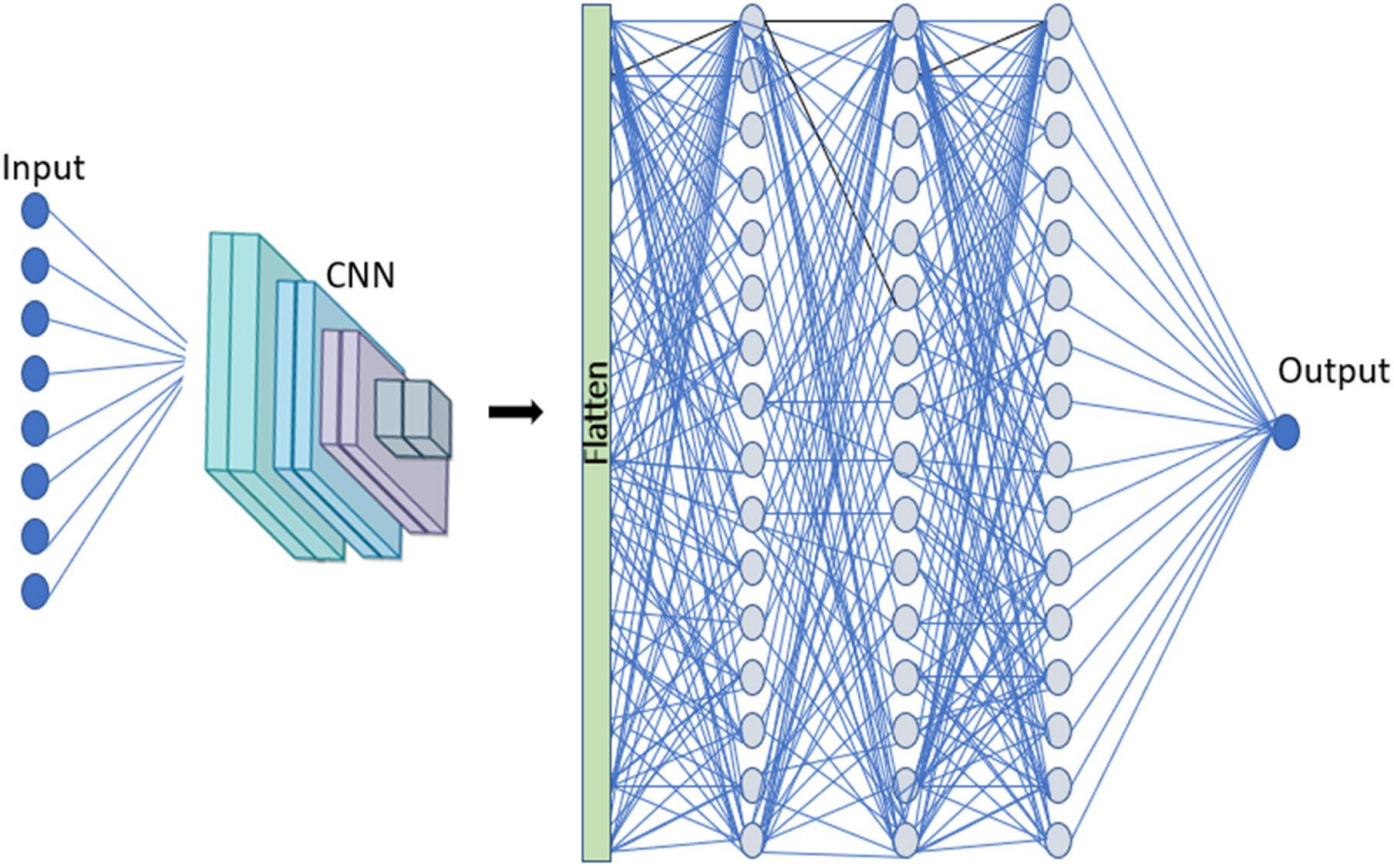
Hybrid CNN-ANN Architecture.

The network CNN-ANN combines both networks with the extraction of functionalities. CNN uses kernel technology to upgrade filter weights to understand how the training data are represented. The model contains a single CNN layer with 5 * 2 * 2-stride filters that complement the input data. The model of CNN contains hidden neuronal layers depending on the model for a specific dataset. The output of the CNN layer is flattened so that the complimentary ANN model may be supplied. The ANN network also consists of hidden layers of neurons and a one-node output layer. Both models are formed to compute the relevant derivatives as a single end-to-end network with a loss function as a cross-entropy. Adam optimizer, a learning rate of 0.001, and a training lot size of 512 were used for different epochs. Figure 6 illustrates the architecture of the model. The neurons in this hybrid system can be summed up as a result of the secret layers.

Every layer in a 1-D convolutional neural network mathematically extracts patterns in $G_{i}$, as it pertains to other input variables using Eq. (13) ^21^.

13 $$ h_{{\ddot{y}}}^{k} = f\left( {\left( {W^{k} *x} \right)_{ij} + b_{k} } \right) $$

W^k^ is the kernel weight associated with the k^th^ feature map, *f* represents the activation feature, and * is the operator. Equation (13), where c is the output $h_{{\ddot{y}}}^{k}$., can be rewritten under Eq. (14).

14 $$ q = f\left( {\left( {W^{k} *x} \right)_{ij} + b_{k} } \right) $$

A flattened layer is utilized in the hybrid model to transform the matrix into a unique vector (Eq. (15)), so that the matrix may be adapted to the ANN model input.

15 $$ Z = f\left( q \right) $$

ANN model is used as input for the output of the flattened layer (Z) (Eq. (16)).

16 $$ y\left( x \right) = L\left( {\mathop \sum \limits_{j = 1}^{N} w_{j} \left( p \right).\,Z_{j} \left( p \right) + c} \right) $$

where $y\left( x \right)$ has been predicted $G_{i}$ is the weight which links neurons to the input layer $w_{j} \left( p \right)$, the variable $Z_{j} \left( p \right)$ is the discrete input variable $t$ and the neuronal bias $c$, of the input variable, $L\left( . \right)$ is the hidden transfer function.

### Hybrid CNN-LSTM-ANN architecture

**Figure 7:**
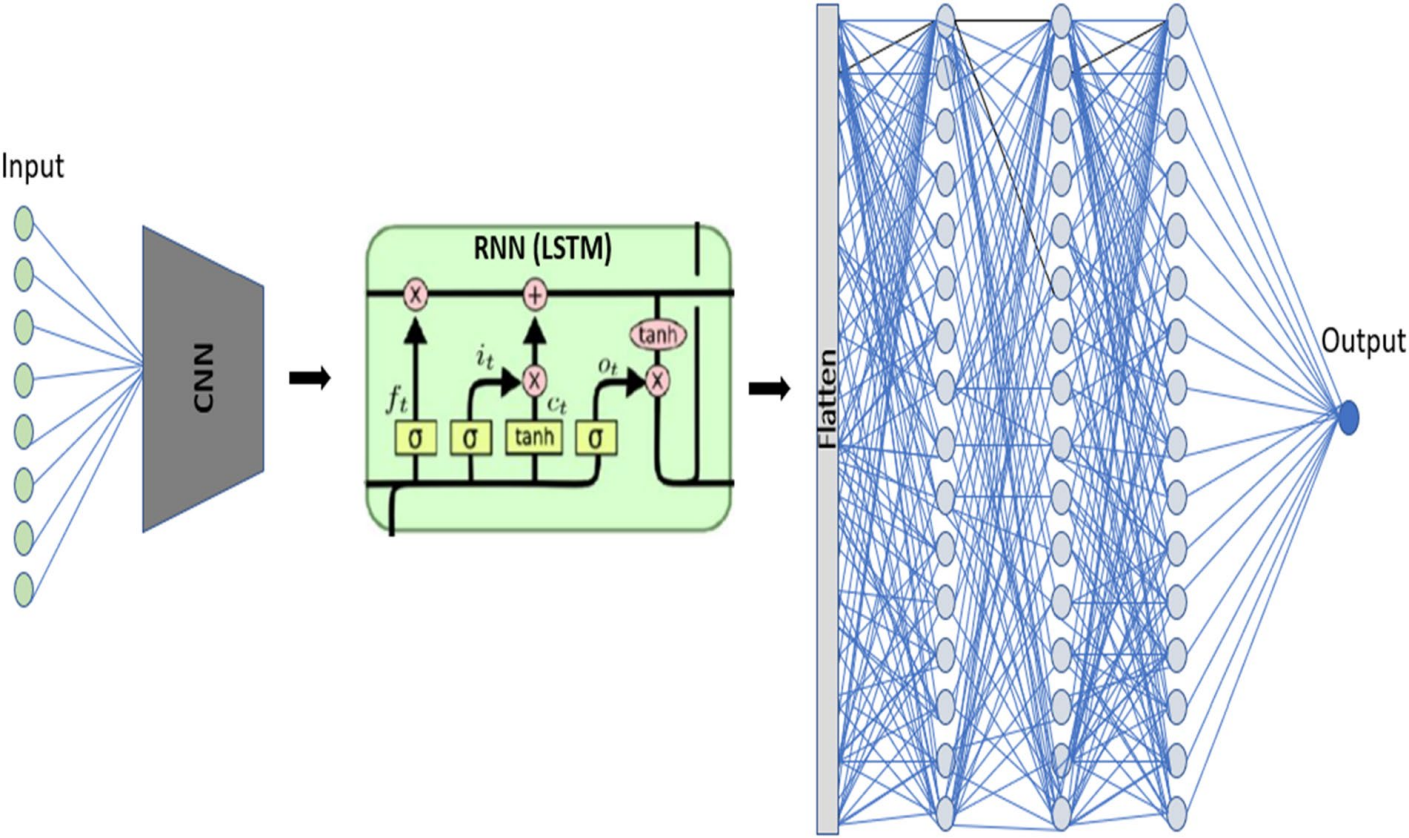
Schematics of the Hybrid CNN-LSTM-ANN Architecture.

The threefold hybrid model has been created to compare the effectiveness of the model in extracting the data by complementing each other in order to understand short and long-term relationships. As shown in Fig. 7, a recurrent neural network is added for this hybrid model which is running in cycles and is extremely proficient in sequence analysis. The combined LSTM helps to maintain the required data from earlier concealed countries compared to the CNN-ANN model. The input data are supplied with neurons to the hidden layer(s) 1D CNN, and then sent to the LSTM network in hidden states and ultimately the densely linked network that generates the overall model forecast. For this hybrid, the ANN model consists of different layers of neurons depending on the data set. The architecture of CNN and ANN is similar to the hybrid CNN-ANN concept mentioned above. This model contains fundamental computations integrated with the synthesis of neurons in the hidden layers of a hybrid model. This is described in four different phases ^78^.

**Phase One:** The LSTM model determines the information that is thrown away from the $f_{t}$ forgotten gate in Eq. (16), according to the hidden state h_t-1,_ and the new input q_t_ is modeled with Eq. (20).

17 $$ f_{t} = \sigma \left( {W_{f} \times \left[ {h_{t - 1} ,q_{t} } \right] + b_{f} } \right) $$

where W_*f*_ is the matrix weight, the logistic sigmoid function is $\sigma \left( \ldots \right)$ and the bias function is $b_{f}$.

**Phase Two:** The information stored in the cell state is chosen in this step. There is also a new cell candidate ($\tilde{C}_{t}$) created by the 'input gate' *i*_*t*_ is likewise scaled.

18 $$ \tilde{C}_{t} = tanh\left( {W_{C} \times \left[ {h_{t - 1} ,q_{t} } \right] + b_{C} } \right) $$

19 $$ i_{t} = \sigma \left( {W_{i} \times \left[ {h_{t - 1} ,q_{t} } \right] + b_{i} } \right) $$

The hyperbolic tangent function in Eq. (18) is Tanh (…).

**Phase Three:** A combination of the earlier cell state *C*_*t-1*_ and $\tilde{C}_{t}$. will update the new cell *C*_*t*_. $f_{t}$ is affected and is also scalable by *i*_*t*_. in the previous cell.

20 $$ C_{t} = f_{t} *C_{t - 1} + i_{t} *\tilde{C}_{t} $$

**Phase Four:** The final step is to divide the output into two stages and define the resulting cell state by creating an $o_{t}$"output gate." The tanh function triggered *C*_*t*_ is filtered by $o_{t}$. The outcome is the desired output $h_{t}$

21 $$ o_{t} = \sigma \left( {W_{o} \times \left[ {h_{t - 1} ,q_{t} } \right] + b_{o} } \right) $$

22 $$ h_{t} = o_{t} \times tanh\left( {C_{t} } \right) $$

The flattening layer transforms the matrix (Eq. (22)) into a single vector for this hybrid model.

23 $$ Z = f\left( {h_{t} } \right) $$

ANN model is used as input for the output of the flattened layer (Z) (Eq. (16)).

## Data Acquisition and Preparation

The solar radiation dataset for this research is collected from three different databases namely; TMY^79^, SARAH ^80^, and WB-ESMAP^81^. These datasets have been measured for different and nine various specific locations within these countries. The specifics (including longitude, elevation, and latitude) of the locations from which these datasets were measured are summarized in Table 2. Since various solar irradiance types are considered in this study, the data timestep for the datasets also varies.

**Table 2:** Location details of research dataset.

Country	Area	Longitude (decimal degree)	Latitude (decimal degree)	Elevation (m)	Timestep_prediction task
Algeria	Tamarasset	4.679	24.072	874	hourly_GSR
Nigeria	Borno	13.427	11.908	308	hourly_DSR
Central African Republic (CAR)	Vakaga	22.508	9.826	494	hourly_GSR
Nigeria	Abuja	7.4913	9.0723	476	daily_DNI
Senegal	Touba	− 15.9196	14.773	37	minutes_ DHI_RSI_, minutes_ GHI_Sil_, minutes_ GHI_pyr_
Nigeria	Akure	5.19	7.25	396	daily_DNI
Egypt	Mut	28.466	24.475	332	hourly_GSR
Senegal	Fatick	− 16.4135	14.3675	8	minutes_ DHI_RSI_, minutes_ GHI_Sil_, minutes_ GHI_pyr_
South Africa (SA)	Northern Cape	20.464	− 29.186	874	hourly_GSR

### Training and testing of the models

The proposed and compared artificial intelligence (AI) models can be trained using different data sizes. While the hourly solar radiation prediction based on TMY considers 12 years of hourly data, 34 years of data is used for daily solar irradiance prediction. For the WB-ESMAP data which considers the prediction of solar irradiance with the timestep being minutes, 2 years of data were used for training/testing and the dataset summary is presented in Table 3. Also, for all the case studies, 90% of the data are used for training while the remaining 10% are the test dataset. The countries considered for the GSR task include Algeria, the Central African Republic (CAR), South Africa (SA), and Egypt. While Nigeria is considered for the daily average DNI task and hourly DSR task, Senegal is the only country considered for DHI_RSI_, GHI_Sil_, and GHI_pyr_ tasks (Table 2).

**Table 3:** Data training and test set summary.

Database	TMY	SARAH	WB-ESMAP
Type of Solar Irradiance	GSR (global beam direct solar irradiance in W/m^2^), DSR (Diffused solar irradiance in W/m^2^)	DNI (daily average solar radiation flux at the surface normal to the direction of the sun Wh/m^2^)	DHI_RSI_ (Diffused Horizontal Irradiance in W/m^2^), GHI_Sil_ (Global Horizontal Irradiance from silicon pyranometer in W/m^2^), GHI_pyr (_Global Horizontal Irradiance from thermopile pyranometer in W/m^2^)
Data timestep	Hourly	Daily average	Minutes
Data size	12 years	34 years	2 years
Data size (100%)	105,192 × 7	12,670 × 4	566,251 × 13
Training dataset (90%)	94,672 × 7	11,401 × 4	509,624 × 13
Test dataset (10%)	10,517 × 7	1266 × 4	56,624 × 13
Input parameters	Year, month, day, hour, sun elevation, ambient temperature, wind speed at 10 m	Year, month, day, sunshine duration	Year, month, day, hour, minute, air temperature, relative humidity, wind speed, wind direction, calculated wind speed, sensor cleaning, precipitation, Barometric pressure

Since the dataset varies based on the database it was extracted from, the input layers of the dataset also differ. For the datasets from all the databases, three input nodes namely year, month, and day are constant. All the AI models designed for the TMY dataset use an input layer of 7 nodes and these nodes represent the input parameters. In addition to the 3 constant nodes for all the datasets, the other TMY input nodes are hour, ambient temperature, wind speed, and sun elevation. Also, the input layer of the models designed for solar irradiance prediction with the SARAH dataset has (1 node in addition to the aforementioned 3 nodes) a total of 4 nodes. The additional node is the daily sunshine duration. Furthermore, the AI models based on the WB-ESMAP dataset consider an input layer with 10 nodes. These nodes (input parameters) are wind speed, wind direction, precipitation, wind speed, air temperature, relative humidity, barometric pressure, and the other constant 3 nodes (Table 3).

### Model implementation and evaluation metrics

Since these AI models are designed for African (developing) countries, the selection of the number of hidden layers and their corresponding neurons were strategically optimized to ensure fast computation, and optimal convergence, and to avoid model over-fitting. All the AI regression models have been built using the Tensorflow and Keras Application Programming Interface (API) and the mean square error (MSE) in Eq. (24) has been adopted as the loss function while (ReLU) is used as the (nonlinear) activation function. For the deep learning models, the feedforward computation is completed, resulting in the model’s predicted value. This value is compared to the ground truth value or label and the loss is computed. Backpropagation is employed to find the derivative of the model parameters and the cost function is minimized using the “Adam” optimizer. All the AI models were implemented in a Python environment (via Jupyter notebook) which runs with a Core i7, 2.20 GHz system with 16 GB RAM, and GTX1060 6 GB Graphics card.

To have the same basis for comparison, the three most common evaluation metrics for numerical AI tasks are adopted in this study to evaluate the performance of all the models. These include root mean square error (RMSE), mean absolute error (MAE), and correlation coefficient (r). These metrics were chosen based on their adoption in (solar radiation prediction) existing works of literature (in developing countries) ^6,30,82^. The mathematical models of the following metrics are:

24 $$ MSE = \frac{1}{N}\mathop \sum \limits_{i = 1}^{N} \left( {G_{i}^{m} - G_{i}^{p} } \right)^{2} $$

25 $$ r = \left( {\frac{{\mathop \sum \nolimits_{i = 1}^{N} \left( {\left( {G_{i}^{m} - \left\langle {G_{i}^{m} } \right\rangle } \right)\left( {G_{i}^{p} - \left\langle {G_{i}^{p} } \right\rangle } \right)} \right)}}{{\sqrt {\mathop \sum \nolimits_{i = 1}^{N} \left( {G_{i}^{m} - \left\langle {G_{i}^{m} } \right\rangle } \right)^{2} } \sqrt {\mathop \sum \nolimits_{i = 1}^{N} \left( {G_{i}^{p} - \left\langle {G_{i}^{p} } \right\rangle } \right)^{2} } }}} \right)^{2} $$

26 $$ MAE = \frac{{\mathop \sum \nolimits_{N}^{t = 1} \left| {G_{i}^{m} - G_{i}^{p} } \right|}}{N} $$

27 $$ RMSE = \sqrt {\frac{1}{N}\mathop \sum \limits_{i = 1}^{N} \left( {G_{i}^{m} - G_{i}^{p} } \right)^{2} } $$

where $$G_{i}^{m}$$ is the measured value and $$G_{i}^{p}$$ represents the predicted value, and $$ <{G_{i}^{m} } >$$/$$< {G_{i}^{p} }>$$ are the average values of $$G_{i}^{m}$$ and $$G_{i}^{p}$$ respectively.

## Results

In this study, the performance of 10 different artificial intelligence models has been compared for various solar irradiance prediction tasks in some selected developing (African countries). While most studies in existing literature have only focused on the hourly forecast of various solar radiation parameters, this study furthers the knowledge in literature by considering different timesteps namely minutes, hourly, and daily. Various solar irradiance parameters (from different measurement techniques) were also considered to highlight the intrinsic attention to detail of the AI models. Considering the technological developmental status of these countries, the models were built to be as simple as possible. In this section performance of all the AI models is discussed. The discussion is presented in three subsections following the timesteps of the solar irradiance parameters.

### Daily average direct normal irradiance prediction

The average daily solar irradiance prediction task considers two locations (namely Akure and Abuja) in Nigeria. Also, the specific solar parameter considered is direct normal solar irradiance (DNI) and this is integral to the performance/ development of many solar-based technologies. The number of hidden layers (as well as the number of neurons in each hidden layer) in each AI model is summarized in Table 4. Also, the optimal number of training epochs and training batch size for each of the models are presented in the same table. This highlights the simplicity of these models and their adaptability to the targeted developing countries.

**Table 4:** Optimal AI training parameters for daily DNI task.

Location	Model	No. of hidden layers, [No. of neurons in each hidden layer]	Batch size	Epoch
Nigeria_Abuja Daily DNI	ANN	3, [***200***, ***200***, ***50***]	128	100
	CNN-ANN	5, [*150*, *150* (), ***150***, ***150***]	512	50
	CNN-LSTM-ANN	3, [*100*, **100**, ***100***]	512	100
	CNN	2, [*150*, *100*]	512	200
	LSTM	2, [**100**, **100**]	512	100
Nigeria_Akure Daily DNI	ANN	3, [***200***, ***200***, ***100***]	128	100
	CNN-ANN	5, [*100*, *32* (), ***100***, ***32***]	512	50
	CNN-LSTM-ANN	3, [*100*, **100**, ***100***]	512	100
	CNN	2, [*150*, *100*]	512	100
	LSTM	2, [**150**, **100**]	512	50

The number of neurons in the hidden layers of the ANN models are written in bold italic; LSTM models in bold; CNN models in italics.

**Figure 8:**
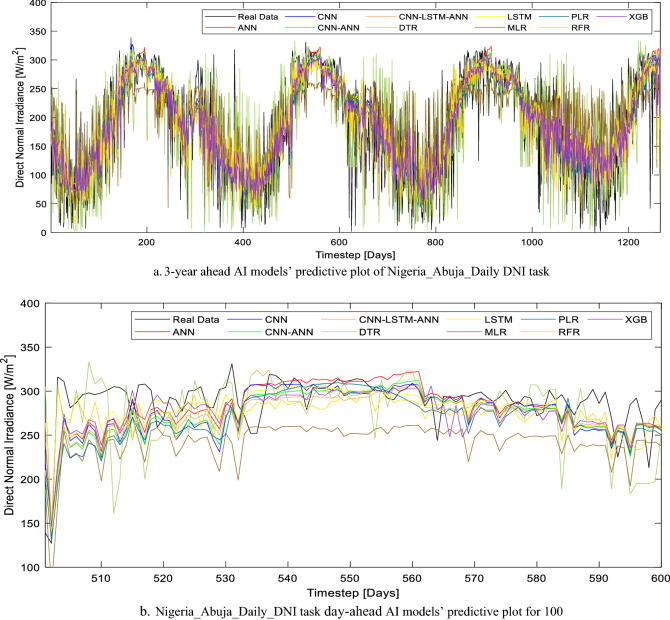
(**a**) 3-year ahead AI models’ predictive plot of Nigeria_Abuja_Daily DNI task. (**b**) Nigeria_Abuja_Daily_DNI task day-ahead AI models’ predictive plot for 100.

Furthermore, the performance of all the models based on the three evaluation metrics used in this study is tabulated in Table 5. Specifically, for Abuja_DNI prediction, two models (DTR and MLR) were found unsuitable for this AI task. This is due to the high RMSE and MAE as well as the low r-value (Table 5). In this study, the models were tasked to forecast the daily average DNI for 3.4 years and the forecasted results in comparison to the real data are compared in Fig. 8a. However, a more detailed pictorial representation (in Fig. 8b) of the forecasted result showed the inadequacies of MLR and DTR. While the performances of ANN, CNN-ANN, and LSTM are quite similar, the most suitable AI models for the Abuja_DNI prediction tasks are CNN-LSTM-ANN and XGB. However, XGB is preferable due to its unsupervised learning characteristics and its fast computational time when compared with CNN-LSTM-ANN.

**Table 5:** Daily DNI task evaluation metric summary.

Location	Model	MAE	RMSE	r
Nigeria_Abuja Daily DNI	ANN	42.69876	55.93012	0.781095
	CNN-ANN	42.37361	55.36583	0.78609
	CNN-LSTM-ANN	41.48851	54.16726	0.79643
	CNN	43.09315	56.52858	0.775707
	DTR	57.92812	75.50730	0.537954
	LSTM	41.90963	55.64409	0.783637
	MLR	56.83012	70.92199	0.610802
	PLR	41.69277	54.27466	0.795517
	RFR	44.44913	58.24596	0.759706
	**XGB**	**40.78282**	**53.73310**	**0.800087**
Nigeria_Akure Daily DNI	ANN	19.10983	25.14591	0.948073
	CNN-ANN	20.09575	26.03551	0.944224
	CNN-LSTM-ANN	19.91106	25.81706	0.945184
	CNN	20.33343	26.26212	0.94322
	DTR	26.03864	34.80059	0.897917
	LSTM	19.78511	25.75553	0.945452
	MLR	21.94447	27.60164	0.937081
	PLR	19.87342	26.03768	0.944214
	RFR	20.42996	26.92031	0.940247
	**XGB**	**18.52771**	**24.68782**	**0.949997**

Significant values are in [bold].

It is also noteworthy that XGB has the least MAE and RMSE (40.78282 W/m^2^ and 53.73310 W/m^2^ respectively) as well as the least r-value (0.800087) as highlighted in Table 5. The new hybrid deep learning CNN-LSTM-ANN model presented in this study is a viable alternative to XGB as the performance of this model differs slightly. While the CNN-LSTM-ANN r-value is 0.79643, the RMSE and MAE are 41.48851 W/m^2^ and 24.68782 W/m^2^ respectively. The close proximity of this model results (forecasted DNIs) to that of the real data in Fig. 8b further highlights its potency.

**Figure 9:**
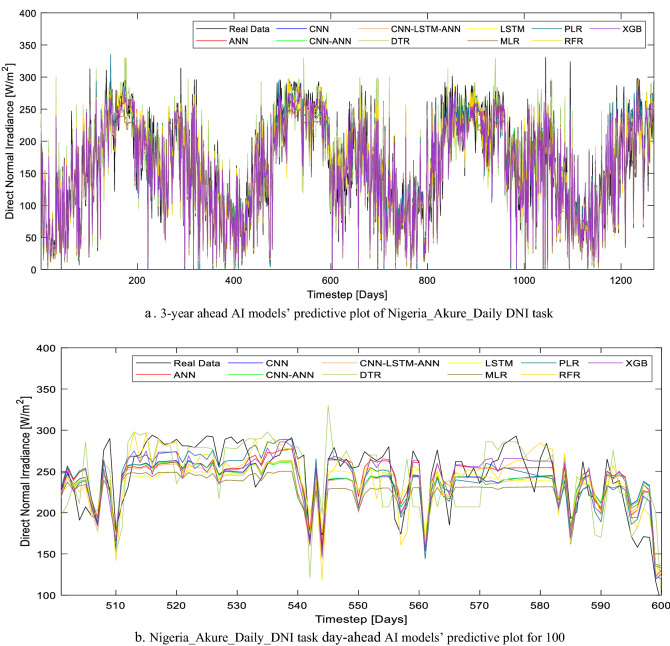
(**a**) 3-year ahead AI models’ predictive plot of Nigeria_Akure_Daily DNI task. (**b**) Nigeria_Akure_Daily_DNI task day-ahead AI models’ predictive plot for 100.

The AI models’ performance for the same task considering another location (Akure_DNI) has a similar pattern to its corresponding Abuja_DNI AI models. Although the only AI model that seems unsuitable for this task is DTR, its performance based on the evaluation metrics is still higher when compared to the Abuja_DNI task (Table 5). The difference in model performance between Abuja_DNI and Akure_DNI prediction tasks can be attributed to the solar distribution in these locations. Akure as a location has a more distributed daily average DNI when compared with Abuja (as seen in Fig. 9a as compared to Fig. 8a), hence the high predictive performance by all the AI models.

While all the models (with the exception of DTR) recorded a good performance for the Akure_DNI prediction task, the best models for this particular task are ANN and XGB. The r-value, RMSE and MAE for these models respectively are 0.948073, 25.14591 W/m^2^, and 19.10983 W/m^2^ for ANN; 0.949997, 24.68782 W/m^2^, and 18.52771 W/m^2^ for XGB. The supervised learning feature of ANN creates room for further improvement of the model (especially when applied in other locations), however, the ANN model overfitting problem should be avoided. As seen in Fig. 9b, the forecasted Akure_DNI with XGB has the closest proximity to the real data. Therefore, it can be inferred that XGB models are most suitable for DNI daily average DNI forecasting.

### Hourly solar radiation forecast

The hourly solar radiation prediction task in this study considers both diffused solar radiation (DSR) and global solar radiation (GSR). The AI models developed for this prediction task are adapted to five locations across Algeria, Nigeria, CAR, Egypt, and South Africa (Table 2). Due to the variation in location, the training parameters for the deep (supervised) learning AI models are optimized to achieve the best predictive performance in each location. Hence, the optimal batch size, number of epochs, number of hidden layers as well as the number of neurons in each hidden layer for all the deep learning models used are highlighted in Table 6.

**Table 6:** Optimal AI training parameters for hourly SR task.

Location	Model	No. of hidden layers, [No. of neurons in each hidden layer]	Batch size	Epoch
Algeria GSR	ANN	3, [***100***, ***100***, ***50***]	512	100
	CNN-ANN	7, [*64*, *64*, *32*, (), ***100***, ***100***, ***50***]	512	100
	CNN-LSTM-ANN	6, [*32*, *32*, **32**, ***50***, ***50***, ***25***]	512	100
	CNN	2, [*150*, *100*]	512	100
	LSTM	2, [**150**, **100**]	512	15
Nigeria DSR	ANN	2, [***100***, ***50***]	512	50
	CNN-ANN	3, [*64*, (), ***50***]	512	30
	CNN-LSTM-ANN	3, [*32*, **32**, ***50***]	512	30
	CNN	2, [*150*, *100*]	512	50
	LSTM	1, [**100**]	512	20
Central African Republic GSR	ANN	3, [***200***, ***200***, ***100***]	512	30
	CNN-ANN	7, [*32*, *64*, *32*, (), ***32***, ***100***, ***32***]	512	10
	CNN-LSTM-ANN	6, [*32*, *16*, **32**, ***25***, ***50***, ***25***]	512	10
	CNN	2, [*150*, *100*]	512	30
	LSTM	1, [**150**]	512	10
Egypt GSR	ANN	3, [***200***, ***200***, ***100***]	128	7
	CNN-ANN	7, [*32*, *64*, *32*, (), ***32***, ***100***, ***32***]	512	10
	CNN-LSTM-ANN	6, [*32*, *16*, **32**, ***25***, ***50***, ***25***]	512	10
	CNN	2, [*150*, *100*]	512	30
	LSTM	2, [**150**, **100**]	512	50
South Africa GSR	ANN	2, [***100***, ***50***]	512	20
	CNN-ANN	3, [*64* (), ***32***]	512	20
	CNN-LSTM-ANN	6, [*32*, *16*, **32**, ***25***, ***50***, ***25***]	512	10
	CNN	2, [*150*, *100*]	512	20
	LSTM	2, [**50**, **50**]	512	50

Significant values are in [bold, italics and bold Italic].

**Figure 10:**
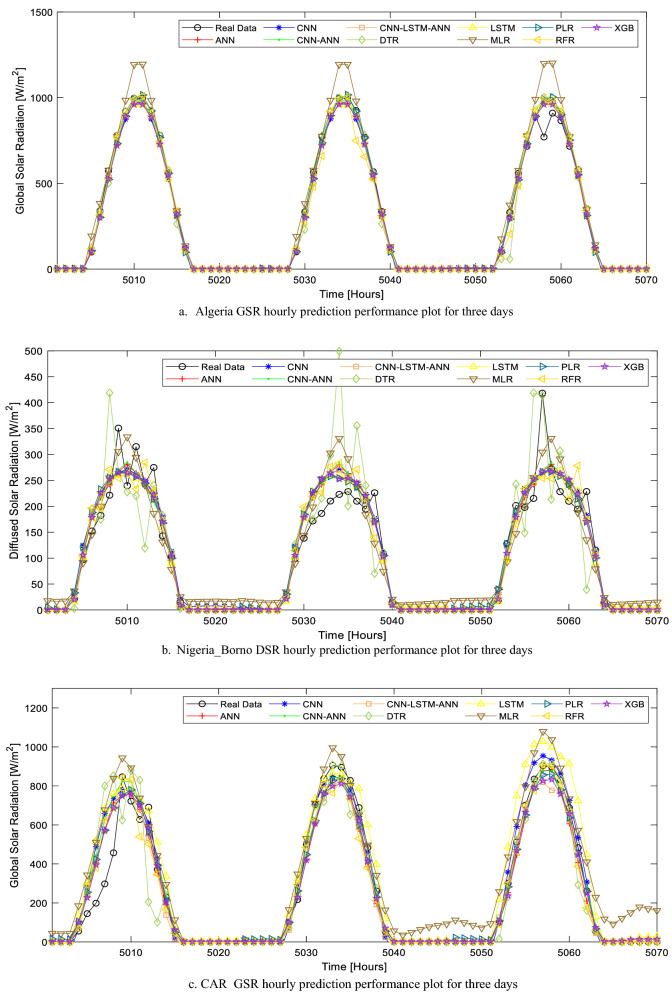

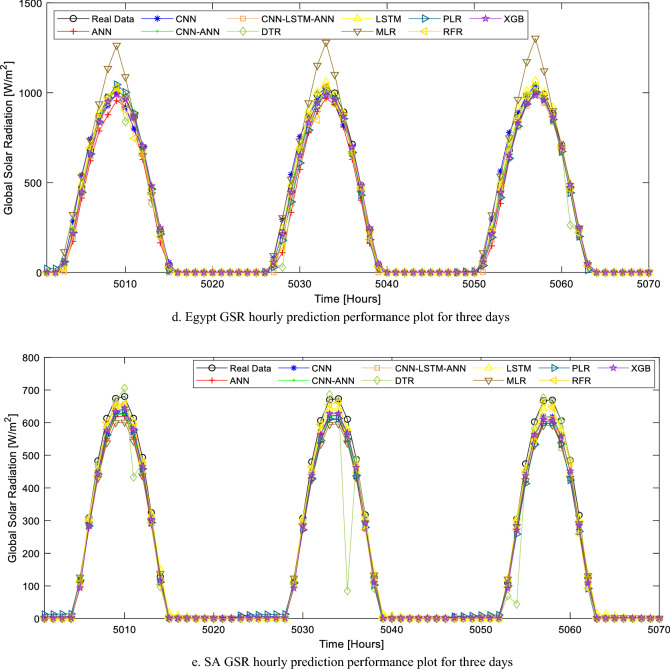
(**a**) Algeria GSR hourly prediction performance plot for three days. (**b**) Nigeria_Borno DSR hourly prediction performance plot for three days. (**c**) CAR GSR hourly prediction performance plot for three days. (**d**) Egypt GSR hourly prediction performance plot for three days. (**e**) SA GSR hourly prediction performance plot for three days.

Out of all the 10 AI models presented in this study, six models have a very good predictive performance on the evaluation metrics results (Table 7). These models are ANN, CNN-ANN, CNN-LSTM-ANN, CNN, PLR, and XGB. The predictive output data (results) in comparison to the real data for all the models over the total test period (for all the location that considers hourly solar radiation forecast) is illustrated (in Fig. A) in the appendix section of this study. From the results of this study, it can also be deduced that the MLR model is not suitable for this specific task (Fig. 10a).

**Table 7:** Hourly SR task evaluation metric summary.

Location	Model	MAE	RMSE	r
Algeria GSR	ANN	27.5867	81.9586	0.977041
	CNN-ANN	28.7015	82.2420	0.976883
	CNN-LSTM-ANN	**30.8785**	**81.1008**	**0.977527**
	CNN	44.2957	85.7817	0.974823
	DTR	42.5385	119.0289	0.950931
	LSTM	41.6829	94.3707	0.969448
	MLR	84.9961	126.1137	0.944743
	PLR	38.4655	84.0446	0.975843
	RFR	35.9412	94.7744	0.96918
	XGB	29.7205	82.0912	0.97697
Nigeria DSR	ANN	19.4431	49.3460	0.904212
	CNN-ANN	18.8024	49.7114	0.902713
	CNN-LSTM-ANN	17.8306	49.8887	0.901976
	CNN	19.0929	49.3699	0.904113
	DTR	25.6896	65.0833	0.826257
	LSTM	18.1817	50.3286	0.900144
	MLR	28.3934	54.5166	0.881686
	PLR	22.9588	51.2770	0.896125
	RFR	19.4016	51.9683	0.893141
	XGB	**17.0214**	**49.1553**	**0.904992**
Central African Republic GSR	ANN	**45.5573**	**95.9444**	**0.965303**
	CNN-ANN	40.5545	97.6806	0.964012
	CNN-LSTM-ANN	44.7466	100.1698	0.962119
	CNN	70.8167	123.9785	0.94135
	DTR	50.0522	133.0457	0.932132
	LSTM	58.4278	118.1977	0.946842
	MLR	90.5368	145.9554	0.917715
	PLR	46.0691	96.4027	0.964966
	RFR	39.9447	100.0757	0.96219
	XGB	40.6753	97.3543	0.964256
Egypt GSR	ANN	26.13274	63.6241	0.986649
	CNN-ANN	62.8158	62.8158	0.986988
	CNN-LSTM-ANN	**22.31752**	**60.49804**	**0.987936**
	CNN	41.16630	72.51108	0.982624
	DTR	24.54221	80.89737	0.978325
	LSTM	28.45273	67.50909	0.984956
	MLR	79.57360	118.2226	0.953111
	PLR	28.32741	63.60174	0.986659
	RFR	20.45168	64.86330	0.98612
	XGB	19.78768	61.42671	0.987561
South Africa GSR	ANN	34.67406	93.49844	0.967236
	CNN-ANN	34.55688	93.20957	0.967441
	CNN-LSTM-ANN	30.73122	92.44526	0.967982
	CNN	33.74673	93.20357	0.967446
	DTR	41.61991	124.9466	0.940689
	LSTM	32.51657	93.07633	0.967536
	MLR	38.40439	95.37698	0.965883
	PLR	48.50556	97.67873	0.964185
	RFR	37.60696	99.28082	0.962978
	XGB	**32.59973**	**91.15934**	**0.968881**

Significant values are in [bold].

The hybrid CNN-LSTM-ANN AI model proposed in this study recorded the best predictive performance for the Algeria_GSR task with an r-value, RMSE, and MAE of 0.977527, 81.101 W/m^2^, and 30.8785 W/m^2^. However, the close proximity of ANN, XGB, and CNN-ANN are evident in their predictive performance over a period of 72 h (Fig. 10a). The performance of the models presented in this study further strengthens existing works of literature in this field as the accuracies are higher than some of the reported results in literature.

Unlike Algeria, the hourly solar radiation prediction task for the location in Nigeria considers diffused solar radiation (DSR). While the r-values of the AI models developed for this task are comparatively smaller than that of the GSR task for other countries, the RMSE and MAE are also smaller. This is due to the statistical and meteorological distribution (as seen in Fig. 10b) of DSR when compared with GSR.

It is also noteworthy that most of the existing works of literature in the domain of solar radiation prediction worked on GSR hourly prediction. Therefore, this study further contributes to the literature as these AI models have been optimized for DSR prediction. While six AI models had high predictive performance when used for the Nigeria_DSR task, XGB is the most superior of all the models. As highlighted in Table 7, the RMSE, MAE, and r-value for the XGB model, when used for the Nigeria_DSR task, are 49.1553 W/m^2^, 17.0214 W/m^2^, and 0.904992. The predicted data for all the AI models are compared with the real data over a period of 72 h and highlighted in Fig. 10b.

The other three countries considered for the solar radiation task in this study are CAR, Egypt, and South Africa. The AI models were developed for GSR hourly prediction tasks in this study and the performance of each of these models is highlighted in Table 7. The models that are suitable for the CAR_GSR task are ANN, CNN-ANN, XGB, and PLR. Considering the evaluation metrics (r = 0.965303, MAE = 45.5573 W/m^2^, RMSE = 95.9444 W/m^2^ in Table 7) and the predictive output data plotted in Fig. 10c, ANN is the most suitable AI model for CAR_GSR forecast task.

It is noteworthy that the high MAE and RMSE values reported in this study for hourly solar radiation are due to the GSR unit. While the unit of GSR in this study is W/m^2^, in most literatures, kW/m^2^ is the unit adopted for GSR, hence the lower MAE and RMSE reported in these studies.

The performance of the AI models for the Egypt_GSR prediction task is the best in this entire study and this is due to the high solar intensity and good solar radiation distribution in the location chosen for this country. As seen in Fig. 10d. and Table 7, the most accurate model for GSR prediction in this location is the proposed CNN-LSTM-ANN model in this study. The r-value, RMSE, and MAE of the model are 0.987936, 60.49804 W/m^2^, and 22.31752 W/m^2^ respectively and these are the best evaluation metrics considering all the AI models for this particular location. Although the performance of XGB is quite similar to the CNN-LSTM-ANN model, the supervised learning nature of the model resulted in a better performance when compared to the XGB model. It is also worth noting that all the deep (supervised) learning models in this study have the capacity to give an accurate prediction of hourly solar radiation.

The last location considered for the GSR prediction (in a developing country context) is in South Africa. The performance (considering the r-value) of all the models (except DTR) is very similar for this location. However, as illustrated in Fig. 10e, the GSR forecast using the XGB model is the closest to the real data. This model had the least RMSE and MAE (91.15934 W/m^2^ and 32.59973 W/m^2^ respectively) as well as the highest r-value (0.968881) as highlighted in Table 7. The locations selected for the hourly solar radiation tasks in this study have been chosen considering data availability and good solar radiation potential. The fast computation speed for all the AI models in this study based on the models’ parameters further showcases their potency in application.

### Solar irradiance prediction based on minutes timestep

One of the outstanding contributions of this present study is the development of AI models to forecast solar irradiance based on minutes timestep. Existing works of literature have majorly focused on the hourly solar irradiance prediction, however, the knowledge of solar irradiance minute by minute will further enhance the estimation of energy production from solar-based technology. Two locations in Senegal have been considered and three different measurement techniques for each location. The optimized training parameters for the deep learning models applied for each task are summarized in Table 8.

**Table 8:** Optimal AI training parameters for minute-ahead solar irradiance task.

Location	Model	No. of hidden layers, [No. of neurons in each hidden layer]	Batch size	Epoch
Sengal_Touba_DHI_RSI_	ANN	2, [***50*** (***0.25***), ***50*** (***0.25***)]	128	20
	CNN-ANN	2, [*100*, (), ***100***]	512	10
	CNN-LSTM-ANN	6, [*32*, *16*, **32**, ***25***, ***50***, ***25***]	512	10
	CNN	2, [*50* (*0.25*), *100* (*0.25*), *50* (*0.25*)]	512	10
	LSTM	2, [**100**, **100**]	512	10
Sengal_Touba_GHI_pyr_	ANN	2, [***100*** (***0.25***), ***200*** (***0.25***)]	128	40
	CNN-ANN	2, [*64*, (), ***64***]	512	50
	CNN-LSTM-ANN	6, [*32*, *16*, **32**, ***25***, ***50***, ***25***]	512	10
	CNN	3, [*50*, *150*, *50*]	512	30
	LSTM	2, [**100**, **50**]	512	15
Sengal_Touba_GHI_Sil_	ANN	2, [***100***, ***50***]	128	30
	CNN-ANN	2, [*100*, (), ***100***]	512	50
	CNN-LSTM-ANN	3, [*64*, **32**, ***50***]	512	15
	CNN	2, [*100*, *100*]	512	100
	LSTM	2, [**100**, **50**]	512	25
Sengal_Fatick_DHI_RSI_	ANN	2, [***100*** (***0.25***), ***50*** (***0.25***)]	128	20
	CNN-ANN	2, [*100*, (), ***50***]	512	15
	CNN-LSTM-ANN	6, [*32*, *16*, **32**, ***25***, ***50***, ***25***]	512	20
	CNN	2, [*150*, (*0.25*) , *100*, (*0.25*)]	512	10
	LSTM	2, [**50**, **50**]	512	10
Sengal_Fatick _GHI_pyr_	ANN	2, [***100***, ***50*** (***0.25***)]	128	50
	CNN-ANN	2, [*64*, (), ***64***]	512	50
	CNN-LSTM-ANN	6, [*32*, *16*, **32**, ***25***, ***50***, ***25***]	512	20
	CNN	3, [*50*, *50*, *50*]	512	35
	LSTM	1, [**150**]	512	10
Sengal_Fatick_GHI_Sil_	ANN	2, [***50*** (***0.25***), ***50*** (***0.25***)]	128	150
	CNN-ANN	2, [*32*, *64*, *32*, (), ***32***, ***100***, ***32***]	512	10
	CNN-LSTM-ANN	3, [*32*, *16*, **32**, ***25***, ***50***, ***25***]	512	20
	CNN	2, [*50*, *50*]	512	20
	LSTM	2, [**50**, **50**]	512	20

Significant values are in [bold, italics and bold Italic].

**Figure 11:**
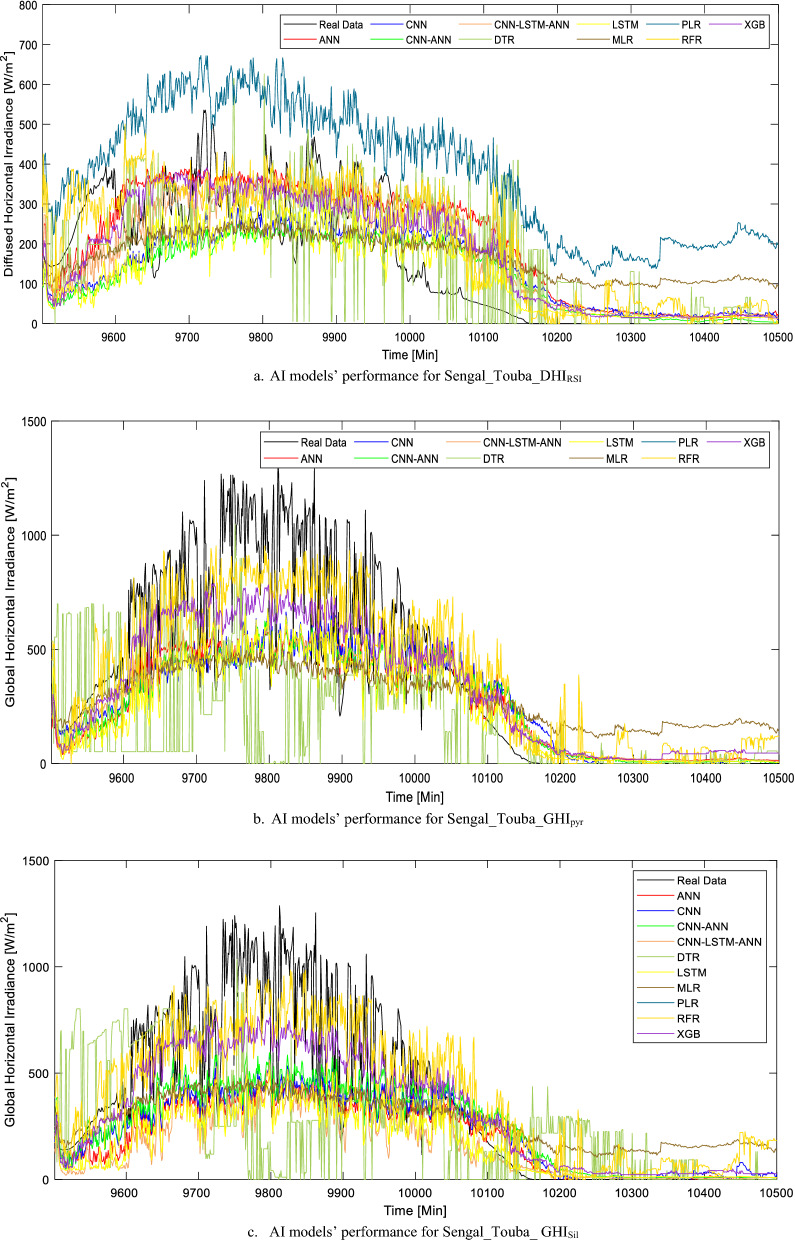
(**a**) AI models’ performance for Sengal_Touba_DHI_RSI_. (**b**) AI models’ performance for Sengal_Touba_GHI_pyr_. (**c**) AI models’ performance for Sengal_Touba_ GHI_Sil_.

One of the things noticed for the preliminary training of all the datasets in this category with the AI models is that the PLR cannot perform this prediction task. Therefore, nine AI models are considered in this section for the solar irradiance prediction task. Generally, the predictive performance of the models (based on the evaluation metrics) shows that it is more difficult for the AI models to accurately forecast solar irradiance minute-by-minute when compared with its corresponding hourly or daily AI models. The nine AI models were tested by using it to forecast the diffused and global horizontal irradiance (DHI_RSI_, GHI_pyr_, and GHI_Sil_) for 39 days in the two locations in Senegal. The forecasted results for Senegal_Toubal are plotted against the actual data and illustrated (in Fig. B) in the Appendix section. However, a day-ahead forecast is also conducted for Senegal_Toubal with the AI models and the results are illustrated in Fig. 11a and b.

Unlike other solar parameters prediction tasks or scenarios in this study (where various models are most suitable for different locations/solar parameters), the training/testing of the solar irradiance in this section showed that the XGB model is the most suitable in all the locations. As seen in Table 8, the AI models have a better performance for DHI_RSI_ and GHI_pyr_ in Senegal_Touba when compared to Senegal_Fatick. While the XGB model performance for DHI_RSI_ forecast task in Sengeal_Touba are r = 0.778685, RMSE = 104.911 W/m^2^, and MAE = 69.41538 W/m^2^, the corresponding best model (XGB) for Senegal_Fatick location are r = 0.727731, RMSE = 118.5533 W/m^2^, and MAE = 82.44148 W/m^2^ (Table 9). As seen in Fig. 11a, while the CNN-LSTM-ANN, LSTM, and ANN models can learn the data part, the proximity of the forecasted data based on the XGB model is better for most of the minutes in the day-ahead task. The plotted results in Fig. 11b and c further confirm the superiority of the XGB model as it follows the real data pattern.

**Table 9:** Minutes timestep SR task evaluation metric summary.

Location	Model	MAE	RMSE	r
Sengal_Touba_DHI_RSI_	ANN	75.13339	105.4392	0.776129
	CNN-ANN	80.79512	115.8425	0.721139
	CNN-LSTM-ANN	73.47140	108.9503	0.758587
	CNN	79.56680	114.5223	0.728642
	DTR	92.30855	146.9156	0.47752
	LSTM	71.02970	106.7229	0.769829
	MLR	96.01363	123.8949	0.671563
	RFR	78.85214	116.3302	0.718326
	**XGB**	**69.41538**	**104.9111**	**0.778685**
Sengal_Fatick_DHI_RSI_	ANN	86.70663	124.1132	0.696014
	CNN-ANN	88.15069	127.3160	0.676376
	CNN-LSTM-ANN	84.16451	120.8993	0.714697
	CNN	91.46472	127.2874	0.669646
	DTR	108.0009	171.3557	0.131339
	LSTM	88.8493	123.0459	0.702328
	MLR	107.4763	136.7249	0.611829
	RFR	88.37136	127.2384	0.676865
	**XGB**	**82.44148**	**118.5533**	**0.727731**
Sengal_Touba_GHI_pyr_	ANN	124.7351	191.4789	0.818723
	CNN-ANN	124.4422	193.0893	0.815315
	CNN-LSTM-ANN	123.4759	191.9940	0.817638
	CNN	137.6269	199.0598	0.802297
	DTR	146.5347	246.9308	0.67041
	LSTM	123.6072	192.8609	0.815801
	MLR	166.3564	232.1552	0.717882
	RFR	123.6086	193.2241	0.815028
	**XGB**	**115.2459**	**176.2756**	**0.848872**
Sengal_Fatick _GHI_pyr_	ANN	144.4373	206.8764	0.79105
	CNN-ANN	136.5069	201.3097	0.803514
	CNN-LSTM-ANN	146.8447	214.7548	0.772475
	CNN	164.9992	224.3841	0.748159
	DTR	174.1384	288.5589	0.521439
	LSTM	154.4912	217.7879	0.765014
	MLR	185.4277	255.2237	0.656054
	RFR	87.64840	126.4613	0.681721
	**XGB**	**82.16708**	**118.6092**	**0.727427**
Sengal_Fatick_GHI_Sil_	ANN	147.8784	203.4149	0.776738
	CNN-ANN	139.2311	207.3560	0.772362
	CNN-LSTM-ANN	142.4694	206.1089	0.775488
	CNN	168.4718	220.0607	0.73864
	DTR	176.7949	287.1410	0.484625
	LSTM	145.6560	210.7485	0.763697
	MLR	178.4968	241.4011	0.673188
	PLR	-	-	-
	RFR	144.6341	212.2252	0.75985
	**XGB**	**128.0217**	**181.4474**	**0.831304**
Sengal_Touba_GHI_Sil_	ANN	124.4958	188.4823	0.801633
	CNN-ANN	120.0924	188.7467	0.801006
	CNN-LSTM-ANN	119.6005	188.2661	0.802144
	CNN	129.8156	188.5900	0.801378
	DTR	146.2485	243.1785	0.635061
	LSTM	120.0516	188.4371	0.801739
	MLR	156.9229	219.7760	0.717001
	RFR	119.4386	185.9876	0.807473
	**XGB**	**109.5886**	**167.9214**	**0.846365**

Significant values are in [bold].

## Brief summary and discussion

Ten AI models have been used as the basis for developing specific algorithms to forecast solar irradiance parameters in this study. Considering the under-development and economic status of many developing countries, the AI models in this study have been adapted for this solar radiation forecast task in six developing (African) countries. It is worth noting that the applicability and the usefulness of the models are beyond developing countries. While two locations in Nigeria were considered for the daily average DNI task, another location in the same country is considered for the hourly average DSR estimation task. Similarly, two locations in Senegal were considered for the estimation of solar irradiance (DHI_RSI_, GHI_pyr_, and GHI_Sil_) estimation task based on minutes timestep. Also, four locations in different countries have been used for GSR estimation. In summary, a total of 13 solar irradiance estimation tasks were carried out in this study considering 10 AI models for each task.

With the aim to check if there is a universal model for solar parameter estimation in developing countries, the results of this study show that various AI models are suitable for different solar irradiance estimations. However, the deep learning models (ANN, LSTM, and CNN), the hybrid deep learning models (CNN-ANN, and CNN-LSTM-ANN) as well as the XGB model has better predictive performance when compared to other models in most location. The results for the prediction of solar irradiance in minutes showed that XGB is the best model for this task in all the locations considered. Also, despite the change in solar measurement parameters in minutes timestep, the performance of the XGB model was relatively suitable for the task. It is, however, noteworthy that the AI models had the least predictive accuracy when considering the minutes' timesteps.

Similarly, the XGB model is the most suitable model for daily average DNI estimation. While PLR and CNN-LSTM-ANN models had a comparatively good performance for this task, the prediction errors recorded by the XGB models are significantly lower. The daily average DNI estimation further shows the novelty of this study as the performance of the models for the Nigeria_Akure_DNI task is better in comparison to existing works of literature. The evaluation metrics for this specific task are r = 0.949997, RMSE = 24.68782, and MAE = 18.52771.

Deep learning models and XGB models are most suited for the hourly solar radiation task. While the innovative hybrid deep learning model (CNN-LSTM-ANN) proposed in this study is most suitable for GSR prediction in Northern African countries, the XGB model reported the best performance for Nigeria and South Africa. Also, the hourly solar radiation estimation accuracy is very high, hence it dominant in existing solar radiation research.

From this study, it can also be deduced that some AI models are not applicable for some specific solar irradiance tasks. PLR model could not learn any of the minute timestep tasks while DTR models also had a bad predictive performance for daily average DNI task. Therefore, these models can be excluded from these specific tasks in the future as they are machine (unsupervised) learning algorithms.

## Conclusions

Based on the results of this study, all the models presented in this study showed their suitability for various solar irradiance prediction tasks. However, the XGB model can be concluded as the best model for solar irradiance prediction tasks out of all the developed AI algorithms considered that was considered within the scope of this research. This is due to its consistently high performance in all the tasks in the study. Despite the change in location and solar parameters, the XGB model had a relatively high performance/accuracy for all the tasks. While the results of the models in the study are better than some existing works of literature, the accuracy of the forecasted solar irradiance shows that more researches on the use of other AI models (such as reinforcement learning models and the developments of new hybrid AI models) are required.

In the future, more research will focus on the accurate prediction of solar irradiance considering the minutes' timestep. While this is the first study to present this (to the best knowledge of the authors), the estimation of solar irradiance in minutes will further help in forecasting solar technology’s production accurately. Thereby, improving the overall development of the solar energy sector.

## Supplementary Information

Supplementary Information.

## Data Availability

The datasets generated and/or analysed during the current study are available from the corresponding author on reasonable request.

## List of symbols

$\hat{Y}$: Random forest algorithm's predictive value
$n, \,\,N, \,\,and\,\, T_{n} \left( x \right).$: Random forest algorithm's predictive mean values
N: Maximum number of samples
$X$: Number of random forest decision trees in N
Σ: Sigma
$Y$: The goal of the polynomial regression
$x$: Polynomial regression predictor
$\theta_{0}$: Polynomial regression bias
$\theta_{0} , \theta_{1} , \ldots . \theta_{n}$: The weight of the equation of regression
$$n$$: Polynomial degree
$\left( {x_{1} ,x_{2} ,x_{3} ,x_{4} } \right)$: Multiple linear regression independent variables
$\left( {\hat{y}} \right)$: Multiple linear regression dependent variable
$b_{0}$: Multiple linear regression Y-axis cut-off point for the adjusted regression curve
$b_{1} ,b_{2} ,b_{3} ,b_{4}$: Multiple linear regression first variable of guessing $x_{1} ,\,\,x_{2} ,\,\,x_{3} ,\,\,x_{4}$
$\left( {R_{1} , R_{2} ,R_{3} \ldots ..R_{J} } \right)$: Decision tree regression predictor space jth regions
$x_{t}$: Long short-term memory current input
$C_{t}$: Long short-term memory new cell states
$C_{t - 1}$: Long short-term memory predecessor cell states
$h_{t}$: Long short-term memory current cell outputs
$h_{t - 1}$: Long short-term memory preceding cell outputs
$W_{i}$: Long short-term memory sigmoid output
$$\left( {\overset{\lower0.5em\hbox{$\smash{\scriptscriptstyle\smile}$}}{C}_{t} } \right)$$: Long short-term memory information
$b_{i}$: Long short-term memory gate bias
$W_{f}$: Long short-term memory weight matrix
$W_{0} \,\,{\text{and}}\,\,b_{0}$: Long short-term memory weighted matrices of the output gate and LSTM bias respectively
$i and j,\,\,\,a_{i} and b_{i}$: Indexes of the artificial neural network neurons
X and Y: Artificial neural network input neurons
$h_{j}$: Artificial neural network hidden layer
$S$: Artificial neural network activation function
$I$: Convolutional neural network input matrix
$K$: Convolutional neural network 2D filter of size $m to n$
$F$: Convolutional neural network 2D feature map output
$I*K$: Indicates the functioning of the convolutionary layer
W^k^: CNN-ANN kernel weight associated with the Kth feature map
*f*: CNN-ANN activation feature
*: CNN-ANN operator
c: CNN-ANN output
$G_{i}$: CNN-ANN weight which links neurons to the input layer $w_{j} \left( p \right)$
$Z_{j} \left( p \right)$: CNN-ANN discrete input variable $t$ and the neuronal bias $c$, of the input variable
$y\left( x \right)$: CNN-ANN prediction
$L\left( . \right)$: CNN-ANN hidden transfer function
$f_{t}$: CNN-LSTM-ann forgotten gate
h_t-1_: CNN-LSTM-ANN hidden state
q_t_: CNN-LSTM-ANN new input
$\sigma \left( \ldots \right)$: CNN-LSTM-ANN logistic sigmoid function
$b_{f}$: CNN-LSTM-ANN bias function
$\left( {\tilde{C}_{t} } \right)$: CNN-LSTM-ANN new cell candidate
*i*_*t*_: CNN-LSTM-ANN scaling factor
$o_{t}$: CNN-LSTM-ANN output gate
$h_{t}$: CNN-LSTM-ANN desired output
