# Supplementary material for: Comprehensive assessment, review, and comparison of AI models for solar irradiance prediction based on different time/estimation intervals
Source: Sci Rep. 2022 Jun 10;12:9644. doi: 10.1038/s41598-022-13652-w (PMC9187635; doi:10.1038/s41598-022-13652-w)
Supplement: Supplementary file 1 — Supplementary Information. [file 41598_2022_13652_MOESM1_ESM.pdf]

Comprehensive Assessment, Review, And Comparison of AI Models for Solar Irradiance Prediction Based on Different Time/Estimation Intervals

Olusola Bamisile[1], Dongsheng Cai[*1], Ariyo Oluwasanmi[2], Chukwuebuka Ejiyi[2], Chiagoziem C. Ukwuoma[2], Oluwasegun Ojo[3,4], Mustapha Mukhtar[5], Qi Huang[1]

[1]Sichuan Industrial Internet Intelligent Monitoring and Application Engineering Technology Research Centre, Chengdu University of Technology, Chenghua District, Chengdu, Sichuan P.R., China.

[2]School of Software Engineering, University of Electronic Science and Technology of China, Chengdu, Sichuan P.R. China.

[3]IMDEA Networks Institute, Leganes, Madrid, 28918, Spain.

[4]Universidad Carlos III de Madrid, Leganes, Madrid, 28912, Spain.

[5]School of Economics and Management, Guangdong University of Petrochemical Technology, Maoming 525000, China.

*Corresponding Author: caidongsheng@cdut.edu.cn

Appendix

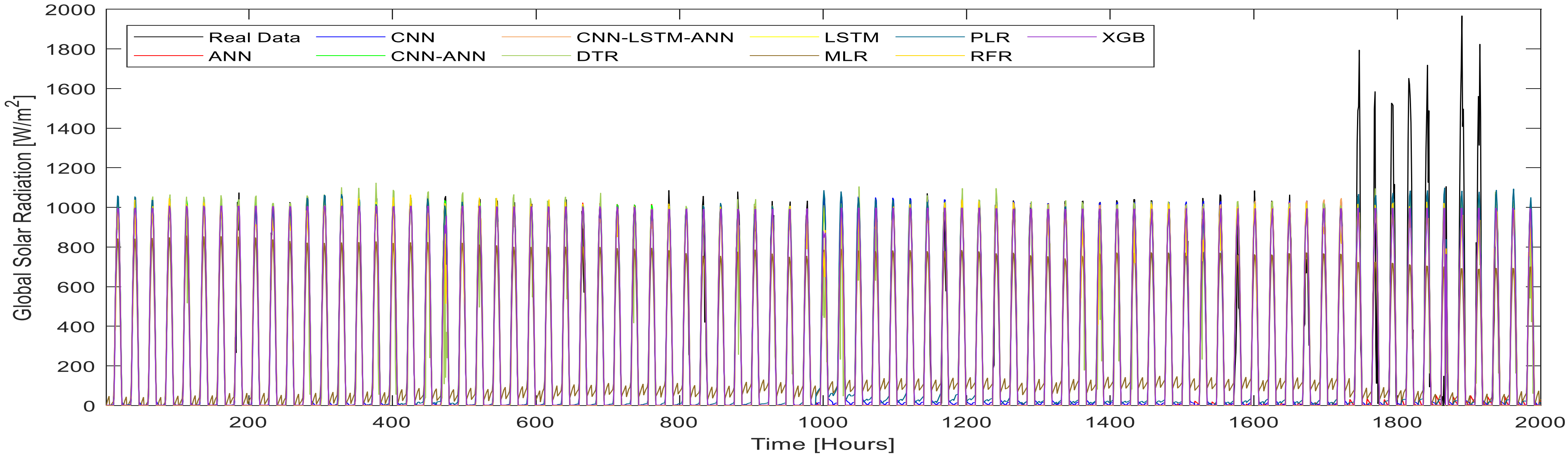

Fig. A1. Algeria GSR hourly prediction performance plot

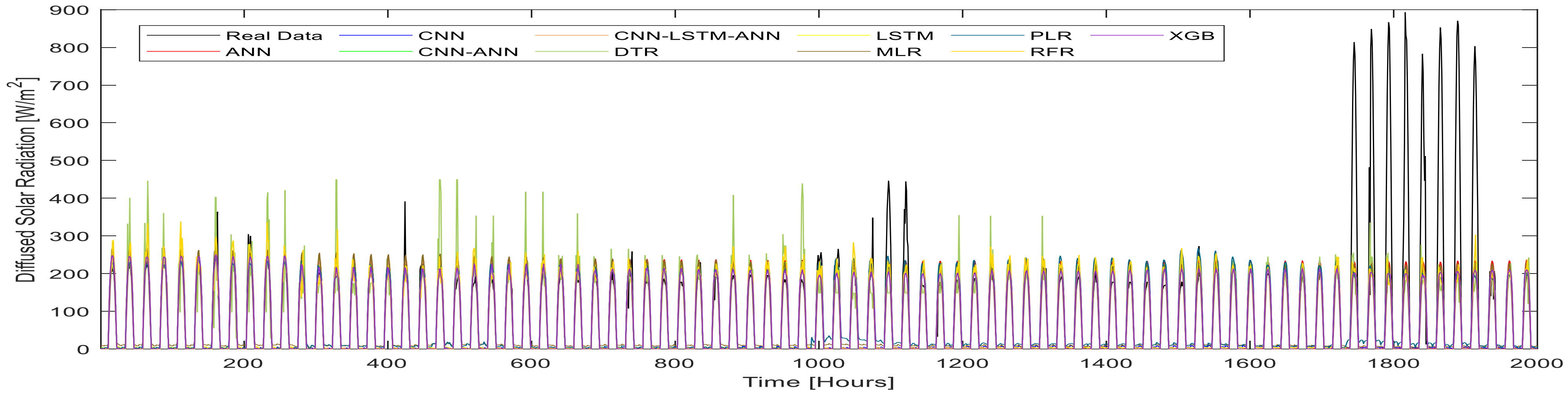

Fig. A2. Nigeria_Born DSR hourly prediction performance plot

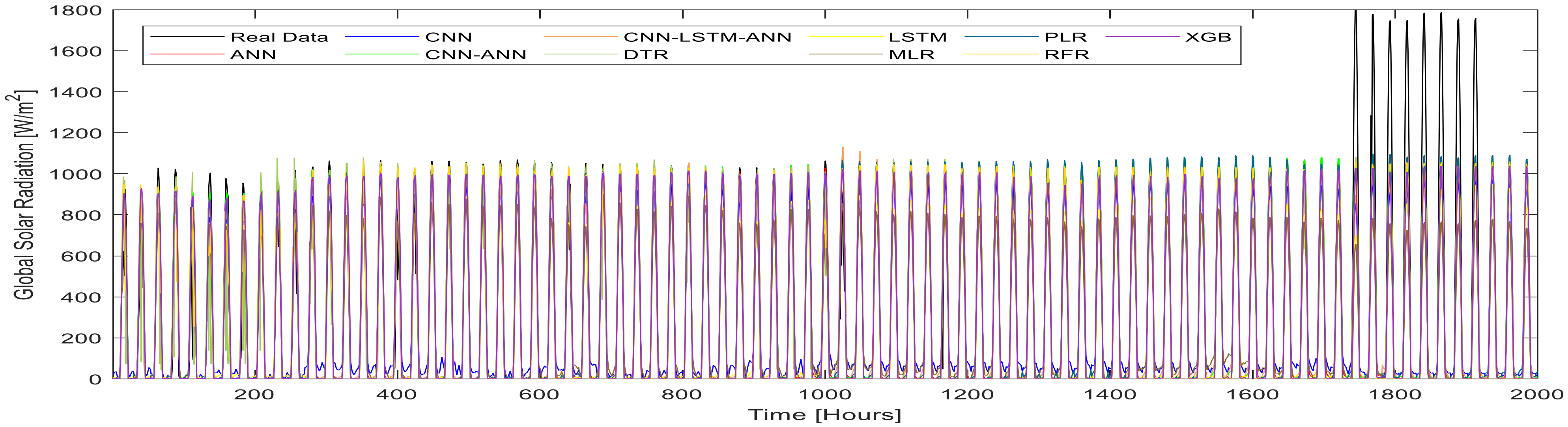

Fig. A3. CAR GSR hourly prediction performance plot

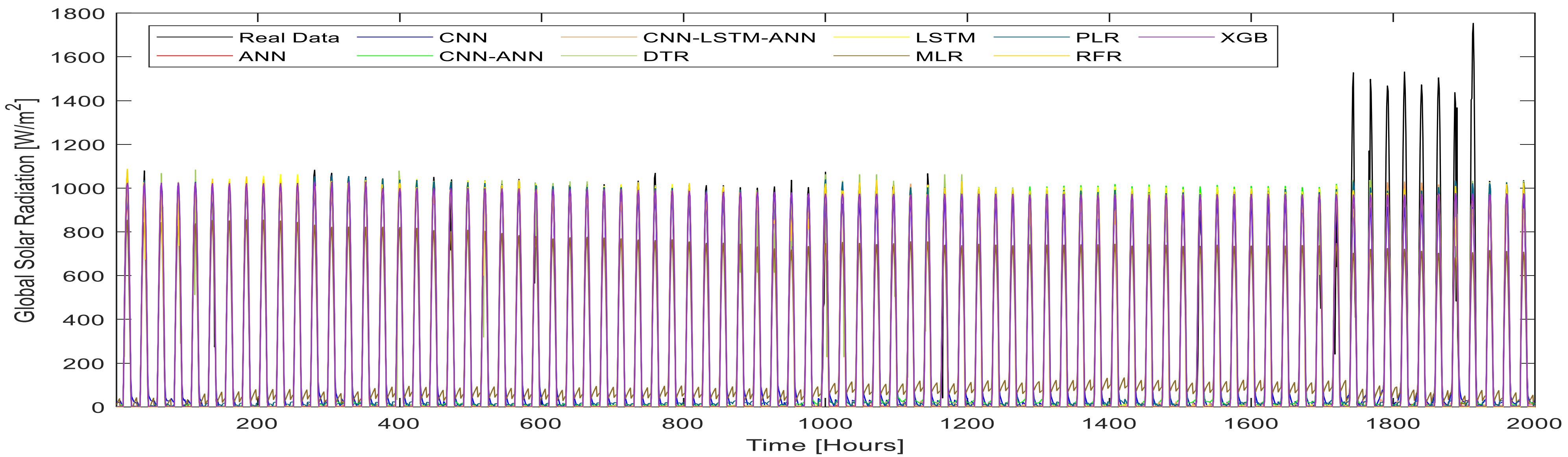

Fig. A4. Egypt GSR hourly prediction performance plot

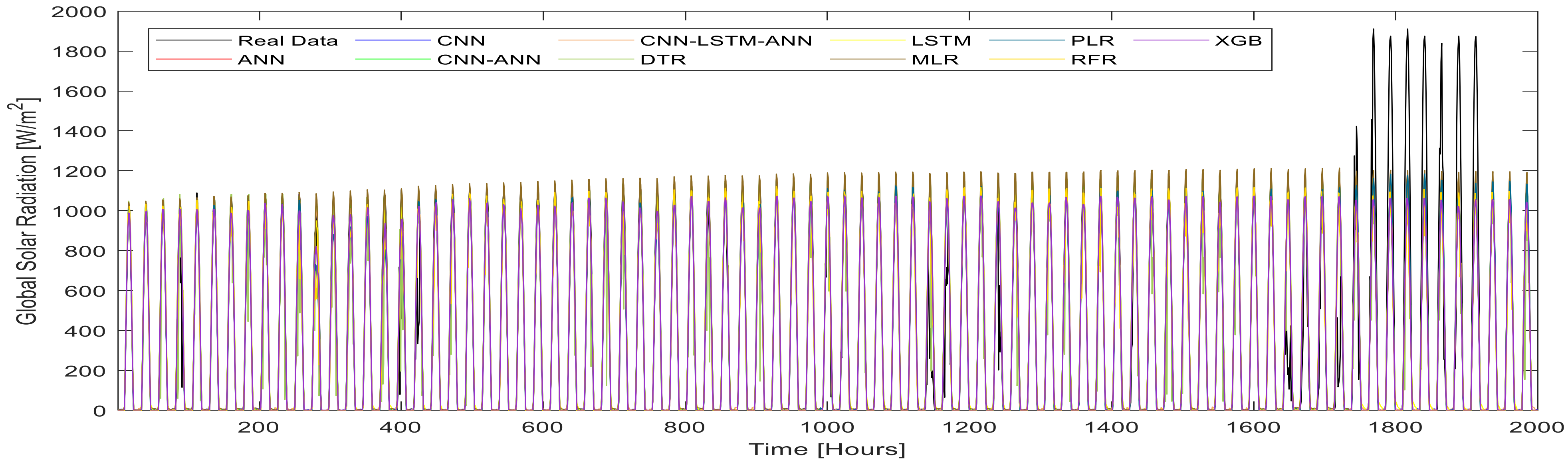

Fig. A5. South Africa GSR hourly prediction performance plot

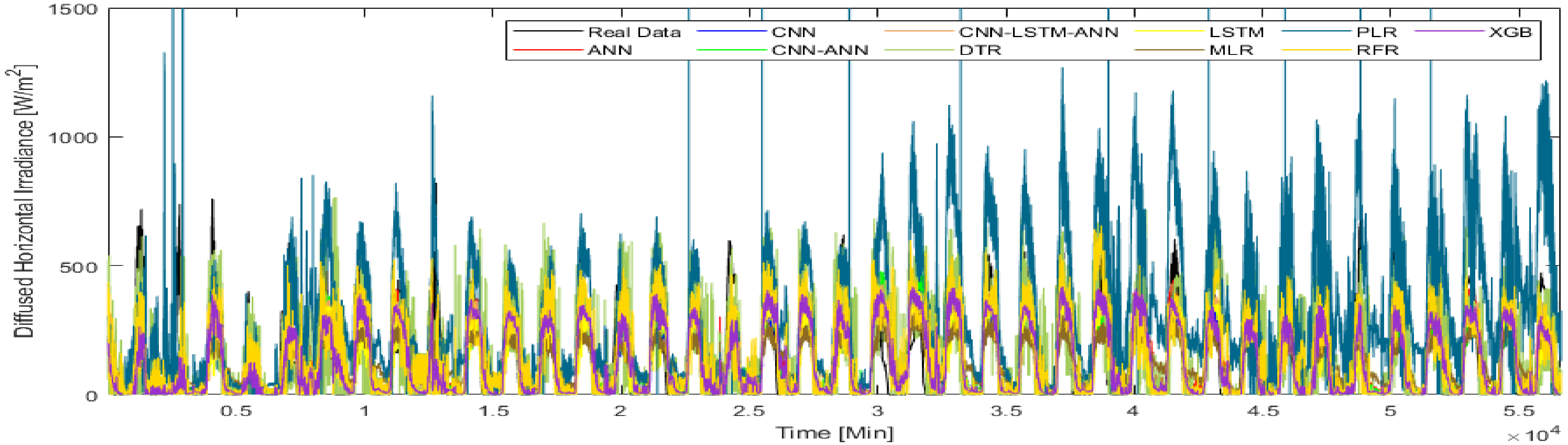

Fig. B1. Performance plot for Sengal_Touba_DHI_{RSI} prediction task

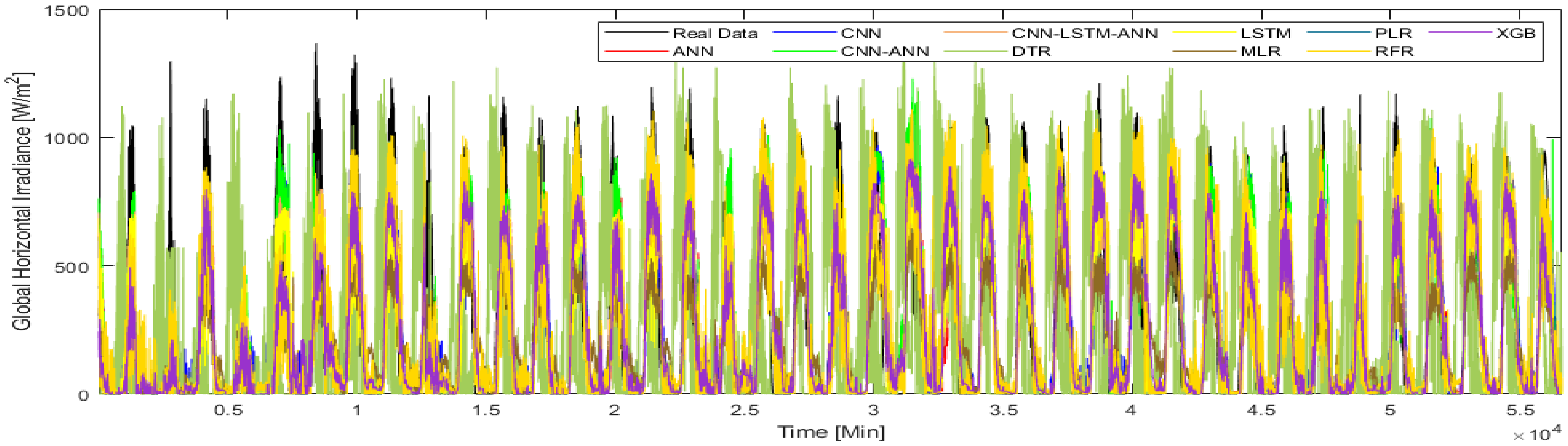

Fig. B2. Performance plot for Sengal_Touba_GHI_{pyr} prediction task

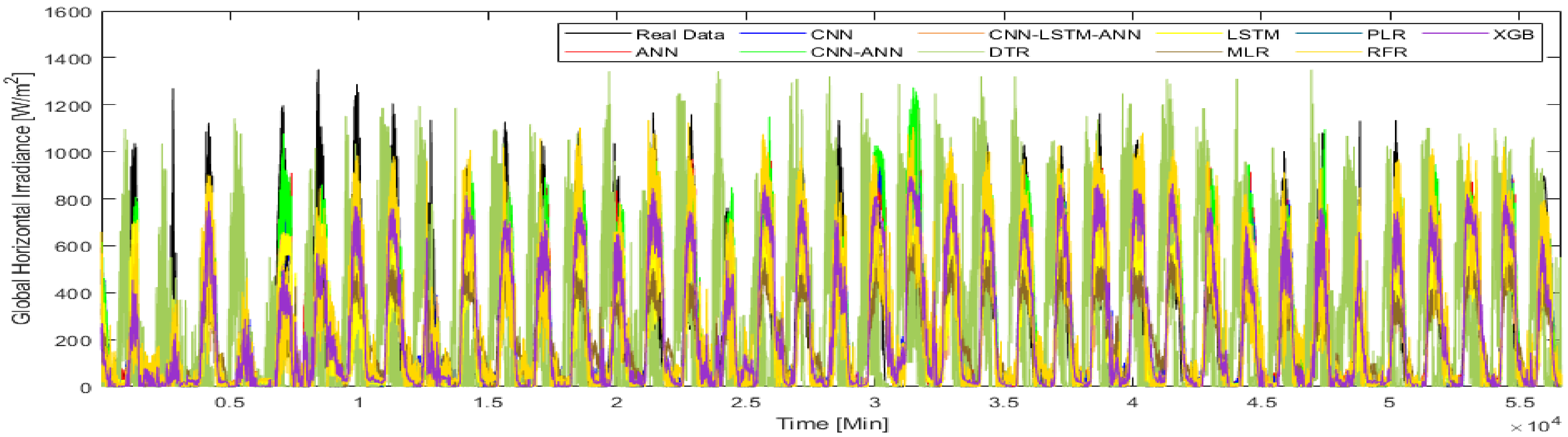

Fig. B3. Performance plot for Sengal_Touba_GHI_{pyr} prediction task
